# Best bang for your buck: GPU nodes for GROMACS biomolecular simulations

**DOI:** 10.1002/jcc.24030

**Published:** 2015-08-04

**Authors:** Carsten Kutzner, Szilárd Páll, Martin Fechner, Ansgar Esztermann, Bert L. de Groot, Helmut Grubmüller

**Affiliations:** ^1^ Theoretical and Computational Biophysics DepartmentMax Planck Institute for Biophysical ChemistryAm Fassberg 1137077GöttingenGermany; ^2^ Theoretical and Computational BiophysicsKTH Royal Institute of Technology17121StockholmSweden

**Keywords:** molecular dynamics, GPU, parallel computing, energy efficiency, benchmark, MD, hybrid parallelization

## Abstract

The molecular dynamics simulation package GROMACS runs efficiently on a wide variety of hardware from commodity workstations to high performance computing clusters. Hardware features are well‐exploited with a combination of single instruction multiple data, multithreading, and message passing interface (MPI)‐based single program multiple data/multiple program multiple data parallelism while graphics processing units (GPUs) can be used as accelerators to compute interactions off‐loaded from the CPU. Here, we evaluate which hardware produces trajectories with GROMACS 4.6 or 5.0 in the most economical way. We have assembled and benchmarked compute nodes with various CPU/GPU combinations to identify optimal compositions in terms of raw trajectory production rate, performance‐to‐price ratio, energy efficiency, and several other criteria. Although hardware prices are naturally subject to trends and fluctuations, general tendencies are clearly visible. Adding any type of GPU significantly boosts a node's simulation performance. For inexpensive consumer‐class GPUs this improvement equally reflects in the performance‐to‐price ratio. Although memory issues in consumer‐class GPUs could pass unnoticed as these cards do not support error checking and correction memory, unreliable GPUs can be sorted out with memory checking tools. Apart from the obvious determinants for cost‐efficiency like hardware expenses and raw performance, the energy consumption of a node is a major cost factor. Over the typical hardware lifetime until replacement of a few years, the costs for electrical power and cooling can become larger than the costs of the hardware itself. Taking that into account, nodes with a well‐balanced ratio of CPU and consumer‐class GPU resources produce the maximum amount of GROMACS trajectory over their lifetime. © 2015 The Authors. Journal of Computational Chemistry Published by Wiley Periodicals, Inc.

## Introduction

Many research groups in the field of molecular dynamics (MD) simulation and also computing centers need to make decisions on how to setup their compute clusters for running the MD codes. A rich variety of MD simulation codes is available, among them CHARMM,^1^ Amber,^2^ Desmond,^3^ LAMMPS,^4^ ACEMD,^5^ NAMD,^6^ and GROMACS.^7^, ^8^ Here, we focus on GROMACS, which is among the fastest ones, and provide a comprehensive test intended to identify optimal hardware in terms of MD trajectory production per investment.

One of the main benefits of GROMACS is its bottom‐up performance‐oriented design aimed at highly efficient use of the underlying hardware. Hand‐tuned compute kernels allow utilizing the single instruction multiple data (SIMD) vector units of most consumer and high performance computing (HPC) processor platforms while OpenMP multithreading and GROMACS’ built‐in thread‐ message passing interface (MPI) library together with nonuniform memory access (NUMA)‐aware optimizations allow for efficient intranode parallelism. Using a neutral‐territory domain‐decomposition (DD) implemented with MPI, a simulation can be distributed across multiple nodes of a cluster. Beginning with version 4.6, the compute‐intensive calculation of short‐range nonbonded forces can be off‐loaded to graphics processing unit (GPUs), while the CPU concurrently computes all remaining forces such as long‐range electrostatics, bonds, so forth, and updates the particle positions.^9^ Additionally, through multiple program multiple data (MPMD) task‐decomposition the long‐range electrostatics calculation can be off‐loaded to a separate set of MPI ranks for better parallel performance. This multilevel heterogeneous parallelization has been shown to achieve strong scaling to as little as 100 particles per core, at the same time reaching high absolute application performance on a wide range of homogeneous and heterogeneous hardware platforms.^10^, ^11^

A lot of effort has been invested over the years in software optimization, resulting in GROMACS being one of the fastest MD software engines available today.^7^, ^12^ GROMACS runs on a wide range of hardware, but some node configurations produce trajectories more economically than others. In this study, we ask: What is the “optimal” hardware to run GROMACS on and how can optimal performance be obtained?

Using a set of representative biomolecular systems, we determine the simulation performance for various hardware combinations, with and without GPU acceleration. For each configuration, we aim to determine the run parameters with the highest performance at comparable numerical accuracy. Therefore, this study also serves as a reference on what performance to expect for a given hardware. Additionally, we provide the GROMACS input files for own benchmarks and the settings that gave optimum performance for each of the tested node types.

Depending on the projects at hand, every researcher will have a somewhat different definition of “optimal,” but one or more of the following criteria C1–C5 will typically be involved:

C1 the performance‐to‐price ratio,
C2 the achievable single‐node performance,
C3 the parallel performance or the “time‐to‐solution,”
C4 the energy consumption or the “energy‐to‐solution,”
C5 rack space requirements.

If on a fixed total budget for hardware, electricity, and cooling, the key task is to choose the hardware that produces the largest amount of MD trajectory for the investment.

Here, we focus on the most suitable hardware for GROMACS MD simulations. Due to the domain‐specific requirements of biomolecular MD and in particular that of algorithms and implementation used by GROMACS, such hardware will likely not be the best choice for a general‐purpose cluster that is intended to serve a broad range of applications. At the same time, it is often possible to pick a middle‐ground that provides good performance both for GROMACS and other applications.

In the next section, we will describe the key determinants for GROMACS performance, and how GROMACS settings can be tuned for optimum performance on any given hardware. Using two prototypic MD systems, we will then systematically derive the settings yielding optimal performance for various hardware configurations. For some representative hardware setups, we will measure the power consumption to estimate the total MD trajectory production costs including electrical power and cooling. Finally, for situations where simulation speed is crucial, we will look at highly parallel simulations for several node types in a cluster setting.

## Key Determinants for GROMACS Performance

GROMACS automatically detects a node's hardware resources such as CPU cores, hardware thread support, and compatible GPUs, at run time. The main simulation tool, mdrun, makes an educated guess on how to best distribute the computational work onto the available resources. When executed on a single node using its integrated, low‐overhead thread‐MPI library, built‐in heuristics can determine essentially all launch configurations automatically, including number of threads, ranks, and GPU to rank assignment, allowing to omit some or all of these options. We use the term “rank” for both MPI processes and thread‐MPI ranks here; both have the same functionality, whereas thread‐MPI ranks can only be used within the same node. Additionally, we use the term “threads” or “threading” to refer to OpenMP threads; each rank may, thus, comprise a group of threads. mdrun optimizes the thread layout for data locality and reuse also managing its own thread affinity settings. Default settings typically result in a fairly good simulation performance, and especially in single‐node runs and on nodes with a single CPU and GPU often optimal performance is reached without optimizing settings manually. However, tuning a standard simulation setup with particle‐mesh Ewald^13^ (PME) electrostatics for optimum performance on a compute node with multiple CPUs and GPUs or on a cluster of such nodes typically requires optimization of simulation and launch parameters. To do this, it is important to understand the underlying load distribution and balancing mechanisms.^14^ The control parameters of these allow optimizing for simulation speed, without compromising numerical accuracy.

### Load distribution and balancing mechanisms

GROMACS uses DD to split up the simulation system into $N_{\text{DD}}={\text{DD}}_{x}\times {\text{DD}}_{y}\times {\text{DD}}_{z}$ initially equally‐sized domains and each of these is assigned to an MPI rank. If dynamic load balancing (DLB) is active, the sizes of the DD cells are continuously adjusted during the simulation to balance any uneven computational load between the domains.

In simulations using PME, MPMD parallelization allows dedicating a group of *N*_PME_ ranks to the calculation of the long‐range (reciprocal space) part of the Coulomb interactions while the short‐range (direct space) part is computed on the remaining *N*_DD_ ranks. A particle‐mesh evaluation is also supported for the long‐range component of the Lennard–Jones potential with the Lennard–Jones PME (LJ‐PME) implementation available as of the 5.0 release.^11^, ^15^ The coarse task‐decomposition based on MPMD allows reducing the number of ranks involved in the costly all‐to‐all communication during three dimensional fast Fourier transformation (3D FFT) needed by the PME computation, which greatly reduces the communication overhead.^7^, ^14^ For a large number of ranks *N*_rank_ >> 8, peak performance is, therefore, usually reached with an appropriate separation *N*_rank_ $=N_{\text{DD}}+N_{\text{PME}}$. The number *N*_PME_ of separate PME ranks can be conveniently determined with the g_tune_pme tool,^1^ which is distributed with GROMACS since version 4.5.

When a supported GPU is detected, the short‐range part of Coulomb and van der Waals interactions are automatically off‐loaded, while the long‐range part, as needed for PME or LJ‐PME, as well as bonded interactions are computed on the CPU. For the PME computation, a fine PME grid in combination with a short Coulomb cutoff results in a numerical accuracy comparable to that of a coarse grid with a large cutoff. Therefore, by increasing short‐range interaction cutoff while also increasing the PME grid spacing, GROMACS can gradually shift computational load between particle–particle (PP) and PME computation when the two are executed on different resources. This is implemented in form of an automated static load‐balancing between CPU and GPU or between PP and PME ranks, and it is performed during the initial few hundreds to thousands of simulation steps.

By default, the GROMACS heterogeneous parallelization uses one GPU per DD cell, mapping each accelerator to a PP rank. If explicit threading parameters are omitted, it also automatically distributes the available CPU cores among ranks within a node by spawning the correct number of threads per rank. Both thread count and order takes into account multiple hardware threads per core with hyperthreading (HT). Using fewer and larger domains with GPU acceleration allows reducing overhead associated to GPU off‐loading like CUDA runtime as well as kernel startup and tail overheads.^2^ Conversely, as the minimum domain size is limited by cutoff and constraint restrictions, using larger domains also ensures that both small systems and systems with long‐range constraints can be simulated using many GPUs. Often, however, performance is improved using multiple domains per GPU. In particular, with more CPUs (or NUMA regions) than GPUs per node and also with large‐core count processors, it is beneficial to reduce the thread count per rank by assigning multiple, “narrower” ranks to a single GPU. This reduces multithreading parallelization overheads, and by localizing domain data reduces cache coherency overhead and intersocket communication. Additional benefits come from multiple ranks sharing a GPU as both compute kernels and transfers dispatched from each rank using the same GPU can overlap in newer CUDA versions.

CPU–GPU and DD load balancing are triggered simultaneously at the beginning of the run, which can lead to unwanted interaction between the two. This can have a detrimental effect on the performance in cases where DD cells are, or as a result of DLB become close in size to the cutoff in any dimension. In such cases, especially with pronounced DD load imbalance, DLB will quickly scale domains in an attempt to remove imbalance reducing the domain sizes in either of the *x*, *y*, or *z* dimensions to a value close to the original buffered cutoff. This will limit the CPU–GPU load‐balancing in its ability to scale the cutoff, often preventing it from shifting more work off of the CPU and leaving the GPUs under‐utilized. Ongoing work aims to eliminate the detrimental effect of this load balancer interplay with a solution planned for the next GROMACS release.

Another scenario, not specific to GPU acceleration, is where DLB may indirectly reduce performance by enforcing decomposition in an additional dimension. With DLB enabled, the DD needs to account for domain resizing when deciding on the number of dimensions required by the DD grid. Without DLB, the same number of domains may be obtained by decomposing in fewer dimensions. Although, decomposition in all three‐dimensions is generally possible, it is desirable to limit the number of dimensions to reduce the volumes communicated. In such cases, it can be faster to switch off DLB, to fully benefit from GPU off‐loading.

### Making optimal use of GPUs

In addition to the load distribution and balancing mechanisms directly controlled by GROMACS, with certain GPU boards additional performance tweaks may be exploited. NVIDIA Tesla cards starting with the GK110 microarchitecture as well as some Quadro cards support a so‐called “application clock” setting. This feature allows using a custom GPU clock frequency either higher or lower than the default value. Typically, this is used as a manual frequency boost to trade available thermal headroom for improved performance, but it can also be used to save power when lower GPU performance is acceptable. In contrast, consumer GPUs do not support application clocks but instead use an automated clock scaling (between the base and boost clocks published as part of the specs). This cannot be directly controlled by the user.

A substantial thermal headroom can be available with compute applications because parts of the GPU board are frequently left underutilized or unutilized. Graphics‐oriented functional units, part of the on‐board GDDR memory, and even the arithmetic units may idle, especially in case of applications relying on heterogeneous parallelization. In GROMACS, the CPU–GPU concurrent execution is possible only during force computation, and the GPU is idle most of the time outside this region, typically for 15–40% of a time step. This leaves enough thermal headroom to allow setting the highest application clock on all GPUs to date (see Fig. 4).

Increasing the GPU core clock rate yields a proportional increase in nonbonded kernel performance. This will generally translate into improved GROMACS performance, but its magnitude depends on how GPU‐bound the specific simulation is. The expected performance gain is highest in strongly GPU‐bound cases (where the CPU waits for results from GPU). Here, the reduction in GPU kernel time translates into reduction in CPU wait time hence improved application performance. In balanced or CPU‐bound cases, the effective performance gain will often be smaller and will depend on how well can the CPU–GPU load‐balancing make use of the increased GPU performance. Note that there is no risk involved in using application clocks; even if a certain workload could generate high enough GPU load for the chip to reach its temperature or power limit, automated frequency throttling will ensure that the limits will not be crossed. The upcoming GROMACS version 5.1 will have built‐in support for checking and setting the application clock of compute cards at runtime.

**Figure 1:**
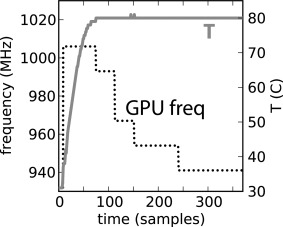
Thermal throttling of the GPU clock frequency on a GeForce GTX TITAN. Starting from a cool, idle state at time *t* = 0, at about *T*  = 36°C, the GPU is put under normal GROMACS load. The clock frequency is first scaled to 1006 MHz, but with the temperature quickly increasing due to the fan speed being capped at the default 60%, the GPU quickly reaches *T* = 80°C, starts throttling, gradually slowing down to 941 MHz.

Indeed, frequency throttling is more common in case of the consumer boards, and factory‐overclocked parts can be especially prone to overheating. Even standard clocked desktop‐oriented GeForce and Quadro cards come with certain disadvantages for compute use. Being optimized for acoustics, desktop GPUs have their fan limited to approximately 60% of the maximum rotation speed. As a result, frequency throttling will occur as soon as the GPU reaches its temperature limit, while the fan is kept at ≤60%. As illustrated on Figure 1, a GeForce GTX TITAN board installed in a well‐cooled rack‐mounted chassis under normal GROMACS workload starts throttling already after a couple of minutes, successively dropping its clock speed by a total of 7% in this case. This behavior is not uncommon and can cause load‐balancing issues and application slowdown as large as the GPU slowdown itself. The Supporting Information shows how to force the GPU fan speed to higher value.

Another feature, available only with Tesla cards, is the CUDA multiprocess server (MPS) which provides two possible performance benefits. The direct benefit is that it enables the overlap of tasks (both kernels and transfers) issued from different MPI ranks to the same GPU. As a result, the aggregate time to complete all tasks of the assigned ranks will decrease. For example, in a parallel run with 2000 atoms per MPI rank, using six ranks and a Tesla K40 GPU with CUDA MPS enabled we measured 30% reduction in the total GPU execution time compared with running without MPS. A secondary, indirect benefit is that in some cases the CPU‐side overhead of the CUDA runtime can be greatly reduced when, instead of the pthreads‐based thread‐MPI, MPI processes are used (in conjunction with CUDA MPS to allow task overlap). Although, CUDA MPS is not completely overhead‐free, at high iteration rates of < 1 ms/step quite common for GROMACS, the task launch latency of the CUDA runtime causes up to 10–30% overhead, but this can be decreased substantially with MPI and MPS. In our previous example using 6‐way GPU sharing, the measured CUDA runtime overhead was reduced from 16 to 4%.

## Methods

We will start this section by outlining the MD systems used for performance evaluation. We will give details about the used hardware and about the software environment in which the tests were done. Then, we will describe our benchmarking approach.

### Benchmark input systems

We used two representative biomolecular systems for benchmarking as summarized in Table 1. The membrane (MEM) system is a membrane channel protein embedded in a lipid bilayer surrounded by water and ions. With its size of ≈80 k atoms, it serves as a prototypic example for a large class of setups used to study all kinds of membrane‐embedded proteins. RIB is a bacterial ribosome in water with ions^16^ and with more than two million atoms an example of a rather large MD system that is usually run in parallel across several nodes.

**Table 1:** Specifications of the two MD systems used for benchmarking.

MD system	Membrane protein (MEM)	Ribosome (RIB)
Symbol used in plots	•	⋆
# particles	81,743	2,136,412
System size (nm)	10.8 × 10.2 × 9.6	31.2 × 31.2 × 31.2
Time step length (fs)	2	4
Cutoff radii^[a]^ (nm)	1.0	1.0
PME grid spacing^[a]^ (nm)	0.120	0.135
Neighborlist update freq. CPU	10	25
Neighborlist update freq. GPU	40	40
Load balancing time steps	5000–10,000	1000–5000
Benchmark time steps	5000	1000–5000

[a] Table lists the initial values of Coulomb cutoff and PME grid spacing. These are adjusted for optimal load balance at the beginning of a simulation.

### Software environment

The benchmarks have been performed with the most recent version of GROMACS 4.6 available at the time of testing (see 5th column of Table 2). Results obtained with version 4.6 will in the majority of cases hold for version 5.0 as the performance of CPU and GPU compute kernels have not changed substantially. Moreover, as long as compute kernel, threading and heterogeneous parallelization design remains largely unchanged, performance characteristics, and optimization techniques described here will translate to future versions, too.^3^

**Table 2:** Overview of the tested node types with the used software stack. These nodes were combined with various GPUs from Table 3

	Hardware per node			Software stack (versions)			
Processor(s)	Total cores	RAM (GB)	IB network	GROMACS	GCC	MPI library^[a]^	CUDA
Intel Core i7‐4770K	4	8	–	4.6.7	4.8.3	–	6.0
Intel Core i7‐5820K	6	16	–	4.6.7	4.8.3	–	6.0
Intel Xeon E3‐1270v2	4	16	QDR	4.6.5	4.4.7	Intel 4.1.3	6.0
Intel Xeon E5‐1620	4	16	QDR	4.6.5	4.4.7	Intel 4.1.3	6.0
Intel Xeon E5‐1650	6	16	QDR	4.6.5	4.4.7	Intel 4.1.3	6.0
Intel Xeon E5‐2670	8	16	QDR	4.6.5	4.4.7	Intel 4.1.3	6.0
Intel Xeon E5‐2670v2	10	32	QDR	4.6.5	4.4.7	Intel 4.1.3	6.0
Intel Xeon E5‐2670v2 × 2	20	32	QDR	4.6.5	4.4.7	Intel 4.1.3	6.0
Intel Xeon E5‐2680v2 × 2	20	64	FDR‐14	4.6.7	4.8.4	IBM PE 1.3	6.5
Intel Xeon E5‐2680v2 × 2	20	64	QDR	4.6.7	4.8.3	Intel 4.1.3	6.0
AMD Opteron 6380 × 2	32	32	–	4.6.7	4.8.3	–	6.0
AMD Opteron 6272 × 4	64	32	–	4.6.7	4.8.3	–	–

[a] For benchmarks across multiple nodes. On single nodes, GROMACS’ built‐in thread‐MPI library was used.

If possible, the hardware was tested in the same software environment by booting from a common software image; on external HPC centers, the provided software environment was used. Table 2 summarizes the hardware and software situation for the various node types. The operating system was Scientific Linux 6.4 in most cases with the exception of the FDR‐14 Infiniband (IB)‐connected nodes that were running SuSE Linux Enterprise Server 11.

For the tests on single nodes, GROMACS was compiled with OpenMP threads and its built‐in thread‐MPI library, whereas across multiple nodes Intel's or IBM's MPI library was used. In all cases, FFTW 3.3.2 was used for computing fast Fourier transformations. This was compiled using −enable‐sse2 for best GROMACS performance.^4^ For compiling GROMACS, the best possible SIMD vector instruction set implementation was chosen for the CPU architecture in question, that is, 128‐bit AVX with FMA4 and XOP on AMD and 256‐bit AVX on Intel processors.

GROMACS can be compiled in mixed precision (MP) or in double precision (DP). DP treats all variables with DP accuracy, whereas MP uses single precision (SP) for most variables, as for example, the large arrays containing positions, forces, and velocities, but DP for some critical components like accumulation buffers. It was shown that MP does not deteriorate energy conservation.^7^ As it produces 1.4–2× more trajectory in the same compute time, it is in most cases preferable over DP.^17^ Therefore, we used MP for the benchmarking.

### GPU acceleration

GROMACS 4.6 and later supports CUDA‐compatible GPUs with compute capability 2.0 or higher. Table 3 lists a selection of modern GPUs (of which all but the GTX 970 were benchmarked) including some relevant technical information. The SP column shows the GPU's maximum theoretical SP flop rate, calculated from the base clock rate (as reported by NVIDIA's deviceQuery program) times the number of cores times two floating‐point operations per core and cycle. GROMACS exclusively uses SP floating point (and integer) arithmetic on GPUs and can, therefore, only be used in MP mode with GPUs. Note that at comparable theoretical SP flop rate the Maxwell GM204 cards yield a higher effective performance than Kepler generation cards due to better instruction scheduling and reduced instruction latencies.

**Table 3:** Some GPU models that can be used by GROMACS. See Figure 4 for how performance varies with clock rate of the Tesla cards, all other benchmarks have been done with the base clock rates reported in this table.

NVIDIA model	Architecture	CUDA cores	Clock rate (MHz)	Memory (GB)	SP throughput (Gflop/s)	≈ Price (€) (net)	
Tesla K20X^[a]^	Kepler GK110	2688	732	6	3935	2800	*✓*
Tesla K40^[a]^	Kepler GK110	2880	745	12	4291	3100	*✓*
GTX 680	Kepler GK104	1536	1058	2	3250	300	*✓*
GTX 770	Kepler GK104	1536	1110	2	3410	320	*✓*
GTX 780	Kepler GK110	2304	902	3	4156	390	*✓*
GTX 780Ti	Kepler GK110	2880	928	3	5345	520	*✓*
GTX TITAN	Kepler GK110	2688	928	6	4989	750	*✓*
GTX TITAN X	Maxwell GM200	3072	1002	12	6156		–
Quadro M6000	Maxwell GM200GL	3072	988	12	6070		Figure 5
GTX 970	Maxwell GM204	1664	1050	4	3494	250	–
GTX 980	Maxwell GM204	2048	1126	4	4612	430	*✓*
GTX 980^+^	Maxwell GM204	2048	1266	4	5186	450	*✓*
GTX 980^‡^	Maxwell GM204	2048	1304	4	5341	450	*✓*

The upper part of the table lists HPC‐class Tesla cards, below are the consumer‐class GeForce GTX cards. Checkmarks (*✓*) indicate which were benchmarked. For the GTX 980 GPUs, cards by different manufacturers differing in clock rate were benchmarked (^+^ and ^‡^ symbols).

As the GROMACS CUDA nonbonded kernels are by design strongly compute‐bound,^9^ GPU main memory performance has little impact on their performance. Hence, peak performance of the GPU kernels can be estimated and compared within an architectural generation simply from the product of clock rate × cores. SP throughput is calculated from the base clock rate, but the effective performance will greatly depend on the actual sustained frequency a card will run at, which can be much higher. At the same time, frequency throttling can lead to performance degradation as illustrated in Figure 1.

The price column gives an approximate net original price of these GPUs as of 2014. In general, cards with higher processing power (Gflop/s) are more expensive; however, the TITAN and Tesla models have a significantly higher price due to their higher DP processing power (1310 Gflop/s in contrast to at most 210 Gflop/s for the 780Ti) and their larger memory. Note that unless an MD system is exceptionally large, or many copies are run simultaneously, the extra memory will almost never be used. For the membrane system (MEM), ≈ 50 MB of GPU memory is needed, and for the ribosome (RIB) ≈ 225 MB. Even an especially large MD system consisting of ≈ 12.5 M atoms uses just about 1200 MB and does, therefore, still fit in the memory of any of the GPUs found in Table 3.

### Benchmarking procedure

The benchmarks were run for 2000–15,000 steps, which translates to a couple of minutes wall clock runtime for the single‐node benchmarks. Balancing the computational load takes mdrun up to a few thousand time steps at the beginning of a simulation. As during that phase the performance is neither stable nor optimal, we excluded the first 1000–10,000 steps from measurements using the ‐resetstep or ‐resethway command line switches. On nodes without a GPU, we always activated DLB, as the benefits of a balanced computational load between CPU cores usually outweigh the small overhead of performing the balancing (see e.g., Fig. 3, black lines). On GPU nodes, the situation is not so clear due to the competition between DD and CPU–GPU load balancing mentioned in the Key Determinants for GROMACS Performance section. We, therefore, tested both with and without DLB in most of the GPU benchmarks. All reported MEM and RIB performances are the average of two runs each, with standard deviations on the order of a few percent (see Fig. 4 for an example of how the data scatter).

#### Determining the single‐node performance

We aimed to find the optimal command‐line settings for each hardware configuration by testing the various parameter combinations as mentioned in the Key Determinants for GROMACS Performance section. On individual nodes with *N*_c_ cores, we tested the following settings using thread‐MPI ranks:

- *N*_rank_ = *N*_c_
- A single process with *N*_th_ = *N*_c_ threads
- Combinations of *N*_rank_ ranks with *N*_th_ threads each, with *N*_rank_ × *N*_th_ = *N*_c_ (hybrid parallelization)
- For *N*_c_ ≥20 without GPU acceleration, we additionally checked with g_tune_pme whether separate ranks for the long‐range PME part do improve performance

For most of the hardware combinations, we checked (a–d) with and without HT, if the processor supports it. The Supporting Information contains a bash script that automatically performs tests (a–c).

To share GPUs among multiple DD ranks, current versions of mdrun require a custom ‐gpu_id string specifying the mapping between PP ranks and numeric GPU identifiers. To obtain optimal launch parameters on GPU nodes, we automated constructing the ‐gpu_id string based on the number of DD ranks and GPUs and provide the corresponding bash script in the Supporting Information.

#### Determining the parallel performance

To determine the optimal performance across many CPU‐only nodes in parallel, we ran g_tune_pme with different combinations of ranks and threads. We started with as many ranks as cores *N*_c_ in total (no threads), and then tested two or more threads per rank with an appropriately reduced number of ranks as in (c), with and without HT.

When using separate ranks for the direct and reciprocal space parts of PME ( $N=N_{\text{DD}}+N_{\text{PME}}$) on a cluster of GPU nodes, only the *N*_DD_ direct space ranks can make use of GPUs. Setting whole nodes aside for the PME mesh calculation would mean leaving their GPU(s) idle. To prevent leaving resources unused with separate PME ranks, we assigned as many direct space (and reciprocal space) ranks to each node as there are GPUs per node, resulting in a homogeneous, interleaved PME rank distribution. On nodes with two GPUs each, for example, we placed *N* = 4 ranks ( $N_{\text{DD}}=N_{\text{PME}}=2$) with as many threads as needed to make use of all available cores. The number of threads per rank may even differ for *N*_DD_ and *N*_PME._ In fact, an uneven thread count can be used to balance the compute power between the real and the reciprocal ranks. On clusters of GPU nodes, we tested all of the above scenarios (a–c) and additionally checked whether a homogeneous, interleaved PME rank distribution improves performance.

## Results

This section starts with four pilot surveys that assess GPU memory reliability (i), and evaluate the impact of compiler choice (ii), neighbor searching frequency (iii), and parallelization settings (iv) on the GROMACS performance. From the benchmark results and the hardware costs, we will then derive for various node types how much MD trajectory is produced per invested €. We will compare performances of nodes with and without GPUs and also quantify the performance dependence on the GPU application clock setting. We will consider the energy efficiency of several node types and show that balanced CPU–GPU resources are needed for a high efficiency. We will show how running multiple simulations concurrently maximizes throughput on GPU nodes. Finally, we will examine the parallel efficiency in strong scaling benchmarks for a selected subset of node types.

### GPU error rates

Opposed to the GeForce GTX consumer GPUs, the Tesla HPC cards offer error checking and correction (ECC) memory. ECC memory, as also used in CPU server hardware, is able to detect and possibly correct random memory bit‐flips that may rarely occur. While in a worst‐case scenario such events could lead to silent memory corruption and incorrect simulation results, their frequency is extremely low.^18^, ^19^ Prior to benchmarking, we performed extensive GPU stress‐tests on a total of 297 consumer‐class GPUs (Table 4) using tools that test for “soft errors” in the GPU memory subsystem and logic using a variety of proven test patterns.^20^ Our tests allocated the entire available GPU memory and ran for ≥ 4500 iterations, corresponding to several hours of wall‐clock time. The vast majority of cards were error‐free, but for eight GPUs, errors were detected. Individual error rates differed considerably from one card to another with the largest rate observed for a 780Ti, where during 10,000 iterations > 50 Million errors were registered. Here, already the first iteration of the memory checker picked up > 1000 errors. On the other end of the spectrum were cards exhibiting only a couple of errors over 10,000 iterations, including the two problematic 980^+^. Error rates were close to constant for each of the four repeats over 10,000 iterations. All cards with detected problems were replaced.

**Table 4:** Frequency of consumer‐class GPUs exhibiting memory errors.

NVIDIA model	GPU memory checker^20^	# Of cards tested	# Memtest iterations	# Cards with errors
GTX 580	MemtestG80	1	10,000	–
GTX 680	MemtestG80	50	4500	–
GTX 770	MemtestG80	100	4500	–
GTX 780	MemtestCL	1	50,000	–
GTX TITAN	MemtestCL	1	50,000	–
GTX 780Ti	MemtestG80	70	4×10,000	6
GTX 980	MemtestG80	4	4×10,000	–
GTX 980^+^	MemtestG80	70	4×10,000	2

### Impact of compiler choice

The impact of the compiler version on the simulation performance is quantified in Table 5. From all tested compilers, GCC 4.8 provides the fastest executable on both AMD and Intel platforms. On GPU nodes, the difference between the fastest and slowest executable is at most 4%, but without GPUs it can reach 20%. Table 5 can also be used to normalize benchmark results obtained with different compilers.

**Table 5:** GROMACS 4.6 single‐node performance with thread‐MPI (and CUDA 6.0) using different compiler versions on AMD and Intel hardware with and without GPUs.

Hardware	Compiler	MEM (ns/d)	RIB (ns/d)	Av. speedup (%)
AMD 6380 × 2	GCC 4.4.7	14	0.99	0
	GCC 4.7.0	15.6	1.11	11.8
	GCC 4.8.3	16	1.14	14.7
	ICC 13.1	12.5	0.96	−6.9
AMD 6380× 2	GCC 4.4.7	40.5	3.04	0
with 2× GTX 980^+^	GCC 4.7.0	38.9	3.09	−1.2
	GCC 4.8.3	40.2	3.14	1.3
	ICC 13.1	39.7	3.09	−0.2
Intel E5–2680v2 × 2	GCC 4.4.7	21.6	1.63	0
	GCC 4.8.3	26.8	1.86	19.1
	ICC 13.1	24.6	1.88	14.6
	ICC 14.0.2	25.2	1.81	13.9
Intel E5–2680v2 × 2	GCC 4.4.7	61.2	4.41	0
with 2× GTX 980^+^	GCC 4.8.3	62.3	4.69	4.1
	ICC 13.1	60.3	4.78	3.5

The last column shows the speedup compared to GCC 4.4.7 calculated from the average of the speedups of the MEM and RIB benchmarks.

### Impact of neighbor searching frequency

With the advent of the Verlet cutoff scheme implementation in version 4.6, the neighbor searching frequency has become a merely performance‐related parameter. This is enabled by the automated pair list buffer calculation based on the maximum error tolerance and a number of simulation parameters and properties of the simulated system including search frequency, temperature, atomic displacement distribution, and the shape of the potential at the cutoff.^9^

**Figure 2:**
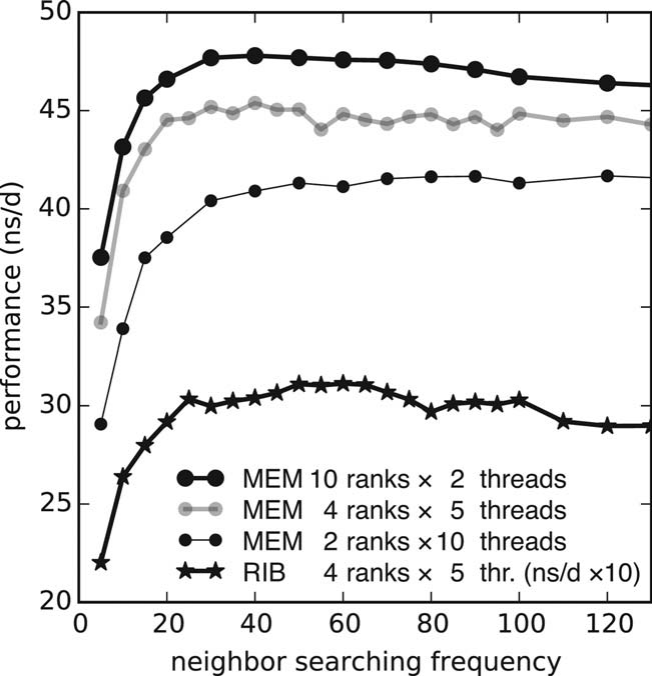
Impact of neighbor searching frequency on the performance on a node with 2×E5‐2680v2 processors and 2×K20X GPUs. In the MEM benchmark the number of ranks and threads per rank was also varied.

Adjusting this frequency allows trading the computational cost of searching for the computation of short‐range forces. As the GPU is idle during list construction on the CPU, reducing the search frequency also increases the average CPU–GPU overlap. Especially in multi‐GPU runs where DD is done at the same step as neighbor search, decreasing the search frequency can have considerable performance impact. Figure 2 indicates that the search frequency optimum is between 20 and 70 time steps. The performance dependence is most pronounced for values ≤20, where performance quickly deteriorates. In our benchmarks, we used a value of 40 on GPU nodes (see Table 1).

**Figure 3:**
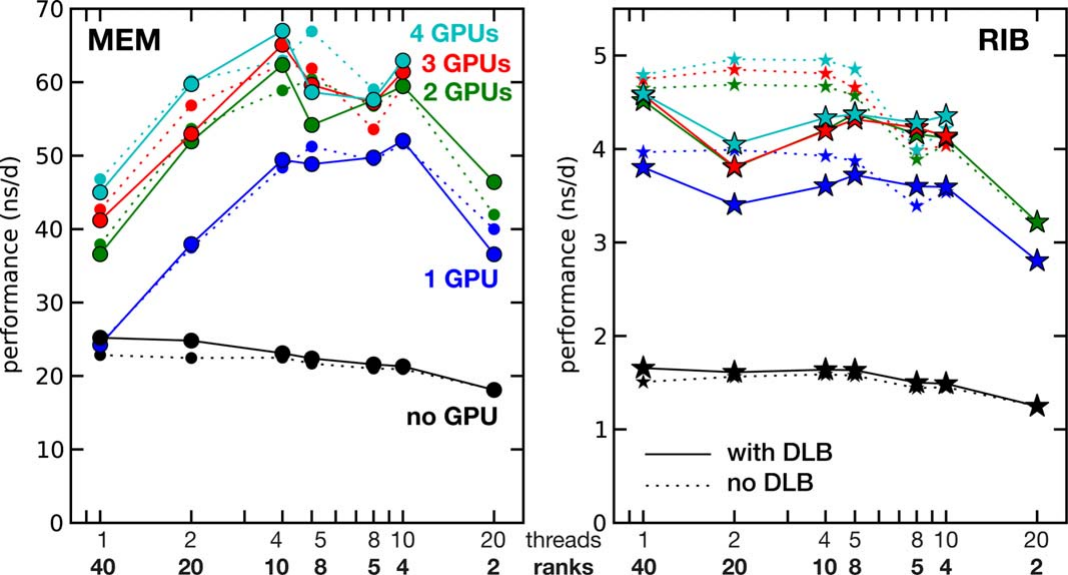
Single‐node performance as a function of the number of GPUs (color coded) and of how the 40 hardware threads are exploited using a combination of MPI ranks and OpenMP threads. Solid lines show performance with, dotted lines without DLB. Test node had 2×E5–2680v2 processors and 4× GTX 980^+^ GPUs. Left panel MEM, right panel RIB benchmark system.

**Figure 4:**
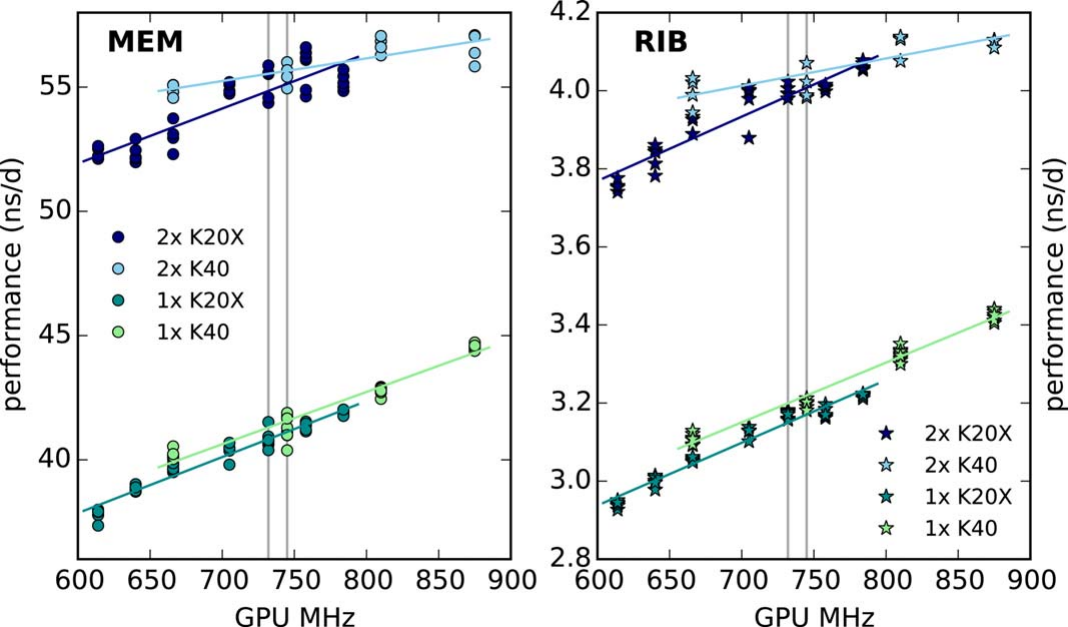
Performance as a function of the GPU application clock rate on a node with 2×E5–2680v2 processors and K20X (dark blue, dark green) or K40 (light blue, light green) GPUs. Gray vertical lines indicate default clock rates. MEM, circles (RIB, stars) benchmarks were run using the settings found in Table 6 (Table 7).

**Figure 5:**
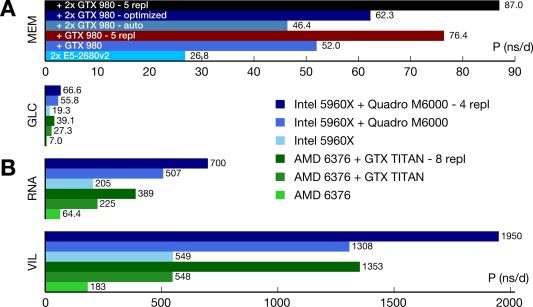
Maximizing throughput by running multiple simulations per node. a) Single‐simulation performance *P* of the MEM benchmark on a node with 2×E5–2680v2 CPUs using 0, 1, or 2 GTX 980^+^ GPUs (blue colors) compared to the aggregated performance of five replicas (red/black). b) Similar to (a), but for different node types and benchmark systems (Available at: http://www.gromacs.org/gpu and ftp://ftp.gromacs.org/pub/CRESTA/CRESTA_Gromacs_benchmarks_v2.tgz). GLC–144 k atoms GluCL CRESTA benchmark, 1 nm cutoffs, PME grid spacing 0.12 nm. RNA–14.7 k atoms solvated RNAse, 0.9 nm cutoffs, PME grid spacing 0.1125 nm. VIL–8 k atoms villin protein, 1 nm cutoffs, PME grid spacing 0.125 nm. In (b), a 5 fs time step and GROMACS 5.0.4 was used. [Color figure can be viewed in the online issue, which is available at wileyonlinelibrary.com.]

**Table 6:** Single‐node performance *P* of the MEM benchmark on various node types.

U	Processor(s) clock rate	CPUs × cores	GPUs	DD		Grid	*N*_PME_	*N*_th_	DLB	*P* (ns/d)	≈ cost (€ net)	ns/d per 205 €
				*x*	*y*	*z*						
D	i7‐4770K	1 × 4	–	2	3	1	2	1	(✓)	7.4	800	1.9
	(3.4‐3.9 GHz)		980^‡^	1	1	1	–	8	–	26.1	1250	4.3
D	i7‐5820K	1 × 6	–	3	3	1	3	1	✓	10.1	850	2.4
	(3.3‐3.6 GHz)		770	1	1	1	–	12	–	26.5	1170	4.6
			980^‡^	1	1	1	–	12	–	32	1390	4.7
1	E3‐1270v2	1 × 4	–	1	1	1	–	8	–	5.3	1080	1
	(3.5 GHz)		680	1	1	1	–	8	–	20	1380	3
			770	1	1	1	–	8	–	20.5	1400	3
1	E5‐1620	1 × 4	680	1	1	1	–	8	–	21	1900	2.3
	(3.6‐3.8 GHz)		770	1	1	1	–	8	–	21.7	1900	2.3
			780	1	1	1	–	8	–	21.8	1970	2.3
			780Ti	1	1	1	–	8	–	23.4	2100	2.3
			TITAN	1	1	1	–	8	–	23.8	2330	2.1
1	E5‐1650	1 × 6	680	1	1	1	–	12	–	22.6	2170	2.1
	(3.2‐3.8 GHz)		770	1	1	1	–	12	–	23.4	2170	2.2
			780	1	1	1	–	12	–	25	2240	2.3
			780Ti	1	1	1	–	12	–	27	2370	2.3
			680 × 2	2	1	1	–	6	(✓)	24.8	2470	2.1
			770 × 2	2	1	1	–	6	(✓)	25.1	2470	2.1
1	E5‐2670	1 × 8	770	1	1	1	–	16	–	26.9	2800	2
	(2.6‐3.3 GHz)		780	1	1	1	–	16	–	28.3	2870	2
			780Ti	1	1	1	–	16	–	29.6	3000	2
			TITAN	1	1	1	–	16	–	29.3	3230	1.9
			770 × 2	2	1	1	–	8	(✓)	27.6	3120	1.8
1	E5‐2670v2	1 × 10	–	4	5	1	–	1	✓	11.2	2480	0.9
	(2.5‐3.3 GHz)		770	1	5	1	–	4	*✗*	29	2800	2.1
			780	1	1	1	–	20	–	29.8	2870	2.1
			780Ti	1	5	1	–	4	(✓)	31.5	3000	2.2
			TITAN	1	1	1	–	20	–	32.7	3230	2.1
			980	1	5	1	–	4	(✓)	33.6	2900	2.4
			770 × 2	10	1	1	–	2	✓	33.7	3120	2.2
			780Ti × 2	10	1	1	–	2	(✓)	35.7	3520	2.1
			980×2	10	1	1	–	2	(✓)	36.8	3330	2.3
4	E5‐2670v2	2 × 10	–	8	5	1	–	1	✓	21.4	3360	1.3
	(2.5‐3.3 GHz)		770	8	1	1	–	5	*✗*	35.9	3680	2
			770 × 2	10	1	1	–	4	✓	51.7	4000	2.6
2			780Ti	8	1	1	–	5	(✓)	45.5	4100	2.3
			780Ti × 2	10	1	1	–	4	✓	56.9	4620	2.5
			780Ti × 3	10	1	1	–	4	(✓)	61.1	5140	2.4
			780Ti × 4	10	1	1	–	4	(✓)	64.4	5660	2.3
2	E5‐2680v2	2 × 10	–	8	2	2	8	1	✓	26.8	4400	1.2
	(2.8‐3.6 GHz)		K20X × 2	8	1	1	–	5	*✗*	55.2	10,000	1.1
			K40 × 2	8	1	1	–	5	*✗*	55.9	10,600	1.1
			980^+^	4	1	1	–	10	✓	52	4850	2.2
			980^+^ × 2	10	1	1	–	4	✓	62.3	5300	2.4
			980^+^ × 3	10	1	1	–	4	✓	65.1	5750	2.3
			980^+^ × 4	8	1	1	–	5	*✗*	66.9	6200	2.2
1	AMD 6272 (2.1)	4 × 16	–	5	5	2	14	1	✓	23.7	3670	1.3
4	AMD 6380	2 × 16	–	5	5	1	7	1	✓	16	2880	1.1
	(2.5 GHz)		TITAN	8	1	1	–	4	*✗*	32.5	3630	1.8
			770 × 2	8	1	1	–	4	✓	35.8	3520	2.1
			980^+^	8	1	1	–	4	*✗*	35.6	3330	2.2
			980^+^ × 2	8	1	1	–	4	*✗*	40.2	3780	2.2

U = rack space requirements in units per node, D for desktop chassis. Prices do not include IB network adapter.

**Table 7:** Same as Table 6, but for the RIB benchmark.

U	Processor(s) clock rate	CPUs × cores	GPUs	DD		Grid	*N*_PME_	*N*_th_	DLB	*P* (ns/d)	≈Cost (€ net)	ns/d per 3600 €
				*x*	*y*	*z*						
D	i7‐4770K	1 × 4	–	2	3	1	–	1	(✓)	0.51	800	2.3
	(3.4‐3.9 GHz)		980^‡^	8	1	1	–	1	*✗*	1.3	1250	3.7
D	i7‐5820K	1 × 6	–	3	3	1	3	1	✓	0.69	850	2.9
	(3.3‐3.6 GHz)		770	4	1	1	–	3	–	1.54	1170	4.7
			980^‡^	1	1	1	–	12	–	1.8	1390	4.7
1	E3‐1270v2	1 × 4	–	1	1	1	–	8	–	0.3	1080	1
	(3.5 GHz)		680	1	1	1	–	8	–	0.89	1380	2.3
			770	1	1	1	–	8	–	0.91	1400	2.3
1	E5‐1620	1 × 4	680	1	1	1	–	8	–	1.03	1900	2
	(3.6‐3.8 GHz)		770	1	1	1	–	8	–	1.02	1900	1.9
			780	1	1	1	–	8	–	1.06	1970	1.9
			780Ti	1	1	1	–	8	–	1.14	2100	2
			TITAN	1	1	1	–	8	–	1.11	2330	1.7
1	E5‐1650	1 × 6	680	1	1	1	–	12	–	1.09	2170	1.8
	(3.2‐3.8 GHz)		770	1	1	1	–	12	–	1.13	2170	1.9
			780	1	1	1	–	12	–	1.17	2240	1.9
			780Ti	1	1	1	–	12	–	1.22	2370	1.9
			680×2	2	1	1	–	6	(✓)	1.4	2470	2
			770×2	2	1	1	–	6	(✓)	1.41	2470	2
1	E5‐2670	1 × 8	770	1	1	1	–	16	–	1.39	2800	1.8
	(2.6‐3.3 GHz)		780	8	1	1	–	2	(✓)	1.6	2870	2
			780Ti	1	1	1	–	16	–	1.64	3000	2
			TITAN	4	1	1	–	4	(✓)	1.67	3230	1.9
			770×2	2	1	1	–	8	(✓)	1.72	3120	2
1	E5‐2670v2	1 × 10	–	4	2	2	4	1	✓	0.79	2480	2.3
	(2.5‐3.3 GHz)		770	10	1	1	–	2	*✗*	1.78	2800	2.3
			780	1	1	1	–	20	–	1.6	2870	2
			780Ti	5	1	1	–	4	(✓)	2.06	3000	2.5
			TITAN	1	1	1	–	20	–	1.75	3230	2
			980	5	1	1	–	4	(✓)	2.22	2900	2.8
			770×2	10	1	1	–	2	*✗*	2.16	3120	2.5
			780Ti×2	4	1	1	–	5	(✓)	2.31	3520	2.4
			980×2	5	1	1	–	4	(✓)	2.34	3330	2.5
4	E5‐2670v2	2 × 10	–	8	2	2	8	1	✓	1.54	3360	1.7
	(2.5‐3.3 GHz)		770	20	1	1	–	2	*✗*	2.71	3680	2.7
			770×2	8	5	1	–	1	*✗*	3.41	4000	3.1
2			780Ti	8	5	1	–	1	(✓)	3.3	4100	2.9
			780Ti×2	8	1	1	–	5	*✗*	4.02	4620	3.1
			780Ti×3	8	5	1	–	1	(✓)	4.17	5140	2.9
			780Ti×4	8	5	1	–	1	(✓)	4.17	5660	2.7
2	E5‐2680v2	2 × 10	–	10	3	1	10	1	✓	1.86	4400	1.5
	(2.8‐3.6 GHz)		K20X×2	20	1	1	–	2	*✗*	3.99	10,000	1.4
			K40×2	20	1	1	–	2	*✗*	4.09	10,600	1.4
			980^+^	20	1	1	–	2	*✗*	3.99	4850	3
			980^+^ × 2	20	1	1	–	2	*✗*	4.69	5300	3.2
			980^+^ × 3	20	1	1	–	2	*✗*	4.85	5750	3
			980^+^ × 4	20	1	1	–	2	*✗*	4.96	6200	2.9
1	AMD 6272 (2.1)	4 × 16	–	5	5	2	14	1	✓	1.78	3670	1.7
4	AMD 6380	2 × 16	–	5	5	1	7	1	✓	1.14	2880	1.4
	(2.5 GHz)		TITAN	16	2	1	–	1	*✗*	2.58	3630	2.6
			770 × 2	16	1	1	–	2	*✗*	2.74	3520	2.8
			980^+^	16	1	1	–	2	*✗*	2.81	3330	3
			980^+^ × 2	16	1	1	–	2	*✗*	3.14	3780	3

### Influence of hybrid parallelization settings and DLB

The hybrid (OpenMP/MPI) parallelization approach in GROMACS can distribute computational work on the available CPU cores in various ways. As the MPI and OpenMP code paths exhibit a different parallel scaling behavior,^10^ the optimal mix of ranks and threads depends on the used hardware and MD system, as illustrated in Figure 3.

For the CPU‐only benchmarks shown in the figure (black lines), pure MPI parallelization yields the highest performance, which is often the case on nodes without GPUs (see fig. 4 in Ref. [ 10]). For multisocket nodes with GPU(s) and for nodes with multiple GPUs, the highest performance is usually reached with hybrid parallelism (with an optimum at about 4–5 threads per MPI rank, colored curves). The performance differences between the individual parallel settings can be considerable: for the single‐GPU setting of the MEM system, for example, choosing 40 MPI ranks results in less than half the performance of the optimal settings, which are four MPI ranks and 10 threads each (24 ns/d compared to 52 ns/d, see blue line in Fig. 3). The settings at the performance optimum are provided in the benchmark summary Tables 6, 7, 11, and 12.

As described in Key Determinants for GROMACS Performance section, especially with GPUs, DLB may in some cases cause performance degradation. A prominent example is the right plot of Figure 3, where the highest RIB performances are recorded without DLB when using GPUs. However, there are also cases where the performance is similar with and without DLB, as for example, in the 4‐GPU case of the left plot (light blue).

### Fitness of various node types

Tables 6 and 7 list single‐node performances for a diverse set of hardware combinations and the parameters that yielded peak performance. “DD grid” indicates the number of DD cells per dimension, whereas “*N*_th_” gives the number of threads per rank. As each DD cell is assigned to exactly one MPI rank, the total number of ranks can be calculated from the number of DD grid cells as *N*_rank_ $={\text{DD}}_{x}\times {\text{DD}}_{y}\times {\text{DD}}_{z}$ plus the number *N*_PME_ of separate PME ranks, if any. Normally, the number of physical cores (or hardware threads with HT) is the product of the number of ranks and the number of threads per rank. For MPI parallel runs, the DLB column indicates whether peak performance was achieved with (symbol ✓) or without DLB (symbol ✗) or whether the benchmark was done exclusively with enabled DLB (symbol (✓)).

The “cost” column for each node gives a rough estimate on the net price as of 2014 and should be taken with a grain of salt. Retail prices can easily vary by 15–20% over a relatively short period. To provide a measure of “bang for buck,” using the collected cost and performance data we derive a performance‐to‐price ratio metric shown in the last column. We normalize with the lowest performing setup to get ≥1 values. While this ratio is only approximate, it still provides insight into which hardware combinations are significantly more competitive than others.

When a single CPU with 4–6 physical cores is combined with a single GPU, using only threading without DD resulted in the best performance. On CPUs with 10 physical cores, peak performance was usually obtained with thread‐MPI combined with multiple threads per rank. When using multiple GPUs, where at least *N*_rank_ $=N_{\text{GPU}}$ ranks is required, in most cases an even larger number of ranks (multiple ranks per GPU) were optimal.

### Speedup with GPUs

Tables 6 and 7 show that GPUs increase the performance of a compute node by a factor of 1.7–3.8. In case of the inexpensive GeForce consumer cards, this also reflects in the node's performance‐to‐price ratio, which increases by a factor of 2–3 when adding at least one GPU (last column). When installing a significantly more expensive Tesla GPU, the performance‐to‐price ratio is nearly unchanged. Because both the performance itself (criterion C2, as defined in the introduction) as well as the performance‐to‐price ratio (C1) are so much better for nodes with consumer‐class GPUs, we focused our efforts on nodes with this type of GPU.

When looking at single‐CPU nodes with one or more GPUs (see third column of Tables 6 and 7), the performance benefit obtained by a second GPU is <20% for the 80 k atom system (but largest on the 10‐core machine), and on average about 25% for the 2 M atom system, whereas the performance‐to‐price ratio is nearly unchanged.

The dual‐GPU, dual‐socket E5‐2670v2 nodes are like the single‐GPU, single‐socket E5‐2670v2 nodes with the hardware of two nodes combined. The dual‐CPU nodes with several GPUs yielded the highest single‐node performances of all tested nodes, up to ≈67 ns/d for MEM and ≈5 ns/d for RIB on the E5‐2680v2 nodes with four GTX 980^+^. The performance‐to‐price ratio (C1) of these 20‐core nodes seems to have a sweet spot at two installed GPUs.

#### GPU application clock settings

For the Tesla K20X and K40 cards, we determined how application clock settings influence the simulation performance (as mentioned previously, GeForce cards do not support manual adjustment of the clock frequency). While the default clock rate of the K20X is 732 MHz, it supports seven clock rates in the range of 614–784 MHz. The K40 defaults to 745 MHz and supports four rates in the range of 666–875 MHz. Application clock rates were set using NVIDIA's system management interface tool. For example, nvidia‐smi ‐ac 2600,875 ‐i 0 sets the GPU core clock rate to 875 MHz and the memory clock rate to 2600 MHz on interface 0.

Figure 4 shows measured performances as a function of the clock rate as well as linear fits (lines). For the K40, the maximum clock rate is about 17% higher than the default, and performance increases about 6.4% when switching from the default to the maximum frequency using a single GPU. The maximum clock rate of the K20X is about 7% higher than the default, resulting in a 2.8% performance increase. For two K40 or K20X GPUs, using the maximum clock rate only results in a 2.1% increased performance, likely because this hardware/benchmark combination is not GPU‐bound. Highest performance is in all cases reached with the GPU application clock set to the highest possible value.

### Energy efficiency

For a given CPU model, the performance/Watts ratio usually decreases with increasing clock rate due to disproportionately higher energy dissipation. CPUs with higher clock rates are, therefore, both more expensive and less energy efficient. On the GPU side, there has been a redesign for energy efficiency with the Maxwell architecture, providing significantly improved performance/Watt compared to Kepler generation cards.

For several nodes with decent performance‐to‐price ratios from Tables 6 and 7, we determined the energy efficiency by measuring the power consumption when running the benchmarks at optimal settings (Tables 8 and 9). On the E5‐2670v2 nodes, we measured the average power draw over an interval of 300 s using a Voltcraft “EnergyCheck 3000” meter. On the E5‐2680v2 nodes, the current energy consumption was read from the power supply with ipmitool.^5^ We averaged over 100 readouts one second apart each. Power measurements were taken after the initial load balancing phase. For the power consumption of idle GPUs, we compared the power draw of idle nodes with and without four installed cards, resulting in ≈27 W (≈24 W) for a single idle 780Ti (980).

**Table 8:** Electric power consumption for nodes with up to four GPUs when running the RIB benchmark.

CPU cores	Installed GPUs	RIB (ns/d)	Power draw (W)	Energy costs (€)	Node costs (€)	5 yr yield (ns/k€)
E5‐2670v2 2 × 10 c. 2.5‐3.3 GHz (GCC 4.4.7)	–	(Node idle)	120	1051	3360	
	–	1.38	252	2208	3360	453
					
	780Ti	3.3	519	4546	3880	714
	780Ti × 2	3.87	666	5834	4400	690
	780Ti × 3	4.17	933	8173	5430	559
	780Ti × 4	4.17	960	8410	5950	530
	980	3.68	408	3574	3780	914
	980 × 2	4.18	552	4836	4200	844
	980 × 3	4.2	696	6097	5130	683
	980 × 4	4.2	840	7358	5550	594
E5‐2680v2 2 × 10 c. 2.8‐3.6 GHz (GCC 4.8.3)	–	(Node idle)	150	1314	4400	
	–	1.86	446	3907	4400	408
					
	980^+^	3.99	6.22	5449	4850	707
	980^+^ × 2	4.69	799	6999	5300	696
	980^+^ × 3	4.85	926	8112	5750	638
	980^+^ × 4	4.96	1092	9566	6200	574

Assuming 5 years of continuous operation and a price of 0.2 € per kWh including cooling, the yield in produced trajectory per invested 1000 € is given in the last column.

**Table 9:** As Table 8, but for the MEM benchmark.

CPU cores	Installed GPUs	MEM (ns/d)	Power draw (W)	Energy costs (€)	Node costs (€)	5‐yr yield (µs/k€)
E5–2680v2 2 × 10 c. 2.8–3.6 GHz (GCC 4.8.3)	–	26.8	446	3907	4400	5.89
					
	980^+^	52.05	547	4792	4850	9.85
	980^+^ × 2	62.34	725	6351	5300	9.77
	980^+^ × 3	65.1	824	7218	5750	9.16
	980^+^ × 4	66.92	899	7875	6200	8.68

**Table 10:** Dependence of simulation performance *P*, cutoff settings, and total power consumption on the graphics processing power for the RIB system on a node with 2 × 2680v2 CPUs and up to 4 GTX 980^+^ GPUs.

Installed GPUs	*P* (ns/d)	Tot. power draw (W)	Cutoff (nm)	Cost ratio short range	Cost ratio PME 3D FFT	Energy efficiency (W/ns/d)
0	1.86	446	1	1	1	240
1	3.99	622	1.16	2.6	0.65	156
2	4.69	799	1.38	3.75	0.46	170
3	4.85	926	1.45	4.22	0.42	191
4	4.96	1092	1.61	5.36	0.36	220

The “cost ratios” indicate the floating point operations in this part of the calculation relative to the CPU‐only case.

**Table 11:** Scaling of the MEM benchmark on different node types with performance *P* and parallel efficiency *E*.

No. of nodes	Processor(s) Intel	GPUs, IB	DD		Grid	*N*_PME_/node	*N*_th_		DLB	*P* (ns/d)	*E*
			*x*	*y*	*z*						
1	E3‐1270v2	770,	1	1	1	–	8	(ht)	–	20.5	1
2	(4 cores)	QDR^[a]^	2	1	1	–	8	(ht)	(✓)	27.2	0.66
4			4	1	1	–	8	(ht)	(✓)	22.1	0.27
8			8	1	1	–	8	(ht)	(✓)	68.3	0.42
16			16	1	1	–	8	(ht)	(✓)	85.7	0.26
32			8	4	1	–	8	(ht)	(✓)	119	0.18
1	E5‐1620	680,	1	1	1	–	8	ht	–	21	1
2	(4 cores)	QDR	2	1	1	–	8	ht	(✓)	29	0.69
4			4	1	1	–	8	ht	(✓)	46.9	0.56
1	E5‐2670v2	780Ti×2,	10	1	1	–	4	ht	✓	56.9	1
2	(2×10 cores)	QDR	4	5	1	–	2		✓	74.2	0.65
4			8	1	1	2	5		*✗*	103.4	0.45
8			8	1	2	2	5		*✗*	119.1	0.26
16			8	4	1	2	5		*✗*	164.8	0.18
32			8	8	1	2	5		*✗*	193.1	0.11
1	E5‐2670v2	980×2,	10	1	1	–	4	ht	(✓)	58	1
2	(2×10 cores)	QDR	4	5	1	–	2		(✓)	75.6	0.65
4			8	5	1	–	2		*✗*	96.6	0.42
1	E5‐2680v2	–	8	2	2	8	1	ht	✓	26.8	1
2	(2×10 cores)	FDR‐14,	4	5	3	10	1	ht	✓	42	0.78
4			8	5	3	10	1	ht	✓	76.3	0.71
8			8	7	2	6	2	ht	✓	122	0.57
16			8	8	4	4	1		✓	162	0.38
32			8	8	8	4	1		✓	209	0.24
64			10	8	6	2.5	2		✓	240	0.14
1	E5‐2680v2	K20X×2	8	1	1	–	5	ht	✓	55.2	1
2	(2×10 cores)	(732 MHz),	4	5	1	–	4	ht	✓	74.5	0.67
4		FDR‐14	8	1	2	–	5		✓	118	0.53
8			8	1	2	2	5		*✗*	163	0.37
16			8	4	1	2	5		*✗*	226	0.26
32			8	8	1	2	5		*✗*	304	0.17

[a] A black “ht” symbol indicates that using all hyperthreading cores resulted in the fastest execution, otherwise using only the physical core count was more advantageous. A gray “(ht)” denotes that this benchmark was done only with the hyperthreading core count (=2 × physical).

Note: These nodes cannot use the full QDR IB bandwidth due to insufficient number of PCIe lanes, see “Strong Scaling” section.

**Table 12:** Same as Table 11, but for the RIB benchmark.

No. of nodes	Processor(s) Intel	GPUs, IB	DD		Grid	*N*_PME_ /node	*N*_th_		DLB	*P* (ns/d)	*E*
			*x*	*y*	*z*						
1	E3‐1270v2	770,	1	1	1	–	8	(ht)	–	0.91	1
2	(4 cores)	QDR^[a]^	2	1	1	–	8	(ht)	(✓)	1.87	1.03
4			4	1	1	–	8	(ht)	(✓)	2.99	0.82
8			8	1	1	–	8	(ht)	(✓)	4.93	0.68
16			16	1	1	–	8	(ht)	(✓)	4.74	0.33
32			16	2	1	–	8	(ht)	(✓)	10.3	0.35
1	E5‐2670v2	780Ti×2,	8	1	1	–	5	(ht)	*✗*	4.02	1
2	(2×10 cores)	QDR	20	1	1	–	4	ht	*✗*	6.23	0.77
4			8	5	1	–	4	ht	*✗*	10.76	0.67
8			16	10	1	–	2	ht	*✗*	16.55	0.51
16			16	10	1	–	2		*✗*	23.78	0.37
32			16	10	2	–	2		*✗*	33.51	0.26
1	E5‐2670v2	980×2,	8	5	1	–	1	ht	(✓)	4.18	1
2	(2×10 cores)	QDR	20	1	1	–	4	ht	*✗*	6.6	0.79
4			8	5	1	–	4	ht	*✗*	11	0.66
1	E5‐2680v2	–	10	3	1	10	1	ht	✓	1.86	1
2	(2×10 cores)	FDR‐14	10	3	1	5	2	ht	✓	3.24	0.87
4			10	2	3	5	2	ht	✓	6.12	0.82
8			8	5	3	5	2	ht	✓	12.3	0.83
16			10	8	3	5	2	ht	✓	21.8	0.73
32			10	7	7	4.69	2	ht	✓	39.4	0.66
64			16	10	6	5	1		✓	70.7	0.59
128			16	16	8	4	1		✓	128	0.54
256			16	17	15	4.06	1		✓	186	0.39
512			20	16	13	1.88	2		*✗*	208	0.22
1	E5‐2680v2	K20X×2	20	1	1	–	2	ht	*✗*	3.99	1
2	(2×10 cores)	(732 MHz),	10	8	1	–	1	ht	*✗*	5.01	0.63
4		FDR‐14	10	8	1	–	2	ht	*✗*	9.53	0.6
8			16	10	1	–	2	ht	*✗*	16.2	0.51
16			16	10	1	–	2		*✗*	27.5	0.43
32			8	8	1	2	5		*✗*	49.1	0.38
64			16	8	1	2	5		*✗*	85.3	0.33
128			16	16	1	2	5		*✗*	129.7	0.25
256			16	8	4	2	5		*✗*	139.5	0.14

[a] Note: These nodes cannot use the full QDR IB bandwidth due to insufficient number of PCIe lanes, see “Strong Scaling” section.

While the total power consumption of nodes without GPUs is lowest, their trajectory costs are the highest due to the very low trajectory production rate. Nodes with one or two GPUs produce about 1.5–2× as much MD trajectory per invested € than CPU‐only nodes (see last column in Tables 8 and 9). While trajectory production is cheapest with one or two GPUs, due to the runs becoming CPU‐bound, the cost rises significantly with the third or fourth card, though it does not reach the CPU‐only level. To measure the effect of GPU architectural change on the energy efficiency of a node, the E5‐2670v2 node was tested both with GTX 780Ti (Kepler) and GTX 980 (Maxwell) cards. When equipped with 1–3 GPUs, the node draws >100 W less power under load using Maxwell generation cards than with Kepler. This results in about 20% reduction of trajectory costs, lowest for the node with two E5‐2670v2 CPUs combined with a single GTX 980 GPU. Exchanging the E5‐2670v2 with E5‐2680v2 CPUs, which have ≈10% higher clock frequency, yields a 52% (44%) increase in energy consumption and 30% (21%) higher trajectory costs for the case of one GPU (two GPUs).

#### Well‐balanced CPU/GPU resources are crucial

With GPUs, the short‐range pair interactions are off‐loaded to the GPU, while the calculation of other interactions like bonded forces, constraints, and the PME mesh, remains on the CPU. To put all available compute power to optimum use, GROMACS balances the load between CPU and GPU by shifting as much computational work as possible from the PME mesh part to the short‐range electrostatic kernels. As a consequence of the off‐load approach, the achievable performance is limited by the time spent by the CPU in the nonoverlapping computation where the GPU is left idle, like constraints calculation, integration, neighbor search, and DD.

Table 10 shows the distribution of the PME and short‐range nonbonded workload with increasing graphics processing power. Adding the first GPU relieves the CPU from the complete short‐range nonbonded calculation. Additionally, load balancing shifts work from the PME mesh (CPU) to the nonbonded kernels (GPU), so that the CPU spends less time in the PME 3D FFT calculation. Both effects yield a 2.1× higher performance compared with the case without a GPU. The benefit of additional GPUs is merely the further reduction of the PME 3D FFT workload (which is just part of the CPU workload) by a few extra percent. The third and fourth GPU only reduce the CPU workload by a tiny amount, resulting in a few percent extra performances. At the same time, the GPU workload is steadily increased, and with it increases the GPU power draw, reflecting in a significantly increased power consumption of the node (see also the other 3‐ and 4‐GPU benchmarks in Table 8).

Hence, GPU and CPU resources should always be chosen in tandem keeping in mind the needs of the intended simulation setup. More or faster GPUs will have little effect when the bottleneck is on the CPU side. The theoretical peak throughput as listed in Table 3 helps to roughly relate different GPU configurations to each other in terms of how much SP compute power they provide and of how much CPU compute power is needed to achieve a balanced hardware setup. At the lower end of GPU models studied here are the Kepler GK104 cards: GTX 680 and 770. These are followed by GK110 cards, in order of increasing compute power, GTX 780, K20X, GTX TITAN, K40, and GTX 780Ti. The GTX 980 and TITAN X, based on the recent Maxwell architecture, are the fastest as well as most power‐efficient GPUs tested. The dual‐chip server‐only Tesla K80 can provide even higher performance on a single board.

The performance‐to‐price ratios presented in this study reflect the characteristics of the specific combinations of hardware setups and workloads used in our benchmarks. Different types of simulations or input setups may expose slightly different ratios of CPU to GPU workload. For example, when comparing a setup using the AMBER force‐field with 0.9 nm cutoffs to a CHARMM setup with 1.2 nm cutoffs and switched van der Waals interactions, the latter results in a larger amount of pair interactions to be computed, hence more GPU workload. This in turn leads to slight differences in the ideal CPU–GPU balance in these two cases. Still, given the representative choices of hardware configurations and simulation systems, our results allow for drawing general conclusions about similar simulation setups.

### Multisimulation throughput

Our general approach in this study is using single‐simulation benchmarks for hardware evaluation. However, comparing the performance *P* on a node with just a few cores to *P* on a node with many cores (and possibly several GPUs) is essentially a strong scaling scenario involving efficiency reduction due to MPI communication overhead and/or lower multithreading efficiency.

This is alleviated by partitioning available processor cores between multiple replicas of the simulated system, which, for example, differ in their starting configuration. Such an approach is generally useful if average properties of a simulation ensemble are of interest. With several replicas, the parallel efficiency is higher, as each replica is distributed to fewer cores. A second benefit is a higher GPU utilization due to GPU sharing. As the individual replicas do not run completely synchronized, the fraction of the time step that the GPU is normally left idle is used by other replicas. The third benefit, similar to the case of GPU sharing by ranks of a single simulation, is that independent simulations benefit from GPU task overlap if used in conjunction with CUDA MPS. In effect, CPU and GPU resources are both used more efficiently, at the expense of getting multiple shorter trajectories instead of a single long one.

Figure 5 quantifies this effect for small to medium MD systems. Subplot A compares the the MEM performance for a single‐simulation (blue colors) to the aggregated performance of five replicas (red/black). The aggregated trajectory production of a multisimulation is the sum of the produced trajectory lengths of the individual replicas. The single simulations settings are found in Table 6; in multisimulations, we used one rank with 40/*N*_rank_ threads per replica. For a single GTX 980, the aggregated performance of a 5‐replica simulation (red bar) is 47% higher than the single simulation optimum. While there is a performance benefit of ≈25% already for two replicas, the effect is more pronounced for ≥4 replicas. For two 980 GPUs, the aggregated performance of five replicas is 40% higher than the performance of a single simulation at optimal settings or 87% higher when compared with a single simulation at default settings (*N*_rank_ = 2, *N*_th_ = 20).

Subplot B compares single and multisimulation throughput for MD systems of different size for an octacore Intel (blue bars) and a 16‐core AMD node (green bars). Here, within each replica we used OpenMP threading exclusively, with the total number of threads being equal to the number of cores of the node. The benefit of multisimulations is always significant and more pronounced the smaller the MD system. It is also more pronounced on the AMD Opteron processor as compared with the Core i7 architecture. For the 8 k atom VIL example, the performance gain is nearly a factor of 2.5 on the 16‐core AMD node.

As with multisimulations one essentially shifts resource use from strong scaling to the embarrassingly parallel scaling regime, the benefits increase the smaller the input system, the larger the number of CPU cores per GPU, and the worse the single‐simulation CPU–GPU overlap.

Section 2.3 in the Supporting Information gives examples of multisimulation setups in GROMACS and additionally quantifies the performance benefits of multisimulations across many nodes connected by a fast network.

### Strong scaling

The performance *P* across multiple nodes is given in Tables 11 and 12 for selected hardware configurations. The parallel efficiency *E* is the performance on *m* nodes divided by *m* times the performance on a single node: $E_{m}=P_{m}/(m\times P_{1})$. In the spirit of pinpointing the highest possible performance for each hardware combination, the multinode benchmarks were done with a standard MPI library, whereas on individual nodes the low‐overhead and therefore faster thread‐MPI implementation was used. This results in a more pronounced drop in parallel efficiency from a single to many nodes than what would be observed when using a standard MPI library throughout. The prices in Tables 6 and 7 do neither include an IB network adapter nor proportionate costs for an IB switch port. Therefore, the performance‐to‐price ratios are slightly lower for nodes equipped for parallel operation as compared to the values in the tables. However, the most important factor limiting the performance‐to‐price ratio for parallel operation is the parallel efficiency that is actually achieved.

The raw performance of the MEM system can exceed 300 ns/d on state‐of‐the‐art hardware, and also the bigger RIB system exceeds 200 ns/d. This minimizes the time‐to‐solution, however, at the expense of the parallel efficiency *E* (last column). Using activated DLB and separate PME ranks yielded the best performance on CPU‐only nodes throughout. With GPUs, the picture is a bit more complex. On large node counts, a homogeneous, interleaved PME rank distribution showed a significantly higher performance than without separate PME ranks. DLB was beneficial only for the MEM system on small numbers of GPU nodes. HT helped also across several nodes in the low‐ to medium‐scale regime, but not when approaching the scaling limit. The performance benefits from HT are largest on individual nodes and in the range of 5–15%.

The E3‐1270v2 nodes with QDR IB exhibit an unexpected, erratic scaling behavior (see Tables 11 and 12, top rows). The parallel efficiency is not decreasing strictly monotonic, as one would expect. The reason could be the CPU's limited number of 20 PCI Express (PCIe) lanes, of which 16 are used by the GPU, leaving only four for the IB adapter. However, the QDR IB adapter requires eight PCIe 2.0 lanes to exploit the full QDR bandwidth. This was also verified in an MPI bandwidth test between two of these nodes (not shown). Thus, while the E3‐1270v2 nodes with GPU offer an attractive performance‐to‐price ratio, they are not well‐suited for parallel operation. Intel's follow‐up model, the E3‐1270v3 provides only 16 PCIe lanes, just enough for a single GPU. For parallel usage, the processor models of the E5‐16x0, E5‐26x0, and E5‐26x0v2 are better suited as they offer 40 PCIe lanes, enough for two GPUs plus IB adapter.

## Discussion

A consequence of off‐loading the short‐ranged nonbonded forces to graphics card(s) is that performance depends on the ratio between CPU and GPU compute power. This ratio can, therefore, be optimized, depending on the requirements of the simulation systems. Respecting that, for any given CPU configuration there is an optimal amount of GPU compute power for most economic trajectory production, which depends on energy and hardware costs.

**Figure 6:**
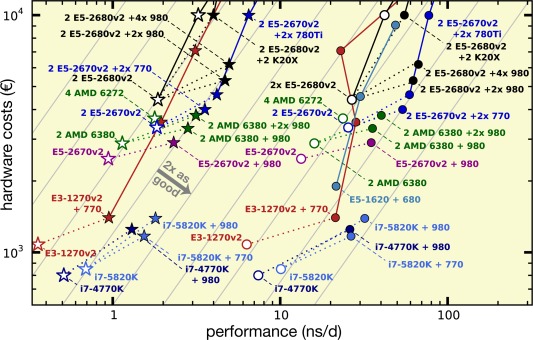
Benchmark performances in relation to the total hardware investment (net) for investments up to 10,000 €. MEM (circles) and RIB (stars) symbols colored depending on CPU type. Symbols with white fill denote nodes without GPU acceleration. Dotted lines connect GPU nodes to their CPU‐only counterparts. The gray lines indicate constant performance‐to‐price ratio, they are a factor of 2 apart each. For this plot, all benchmarks not done with GCC 4.8 (see Table 2) have been renormalized to the performance values expected for GCC 4.8, that is, plus ≈19% for GCC 4.7 benchmarks on CPU nodes and plus ≈4% for GCC 4.7 benchmarks on GPU nodes (see Table 5). The costs for multiple node configurations include 370 € for QDR IB adapters (600 € per FDR‐14 IB adapter) per node. [Color figure can be viewed in the online issue, which is available at wileyonlinelibrary.com.]

**Figure 7:**
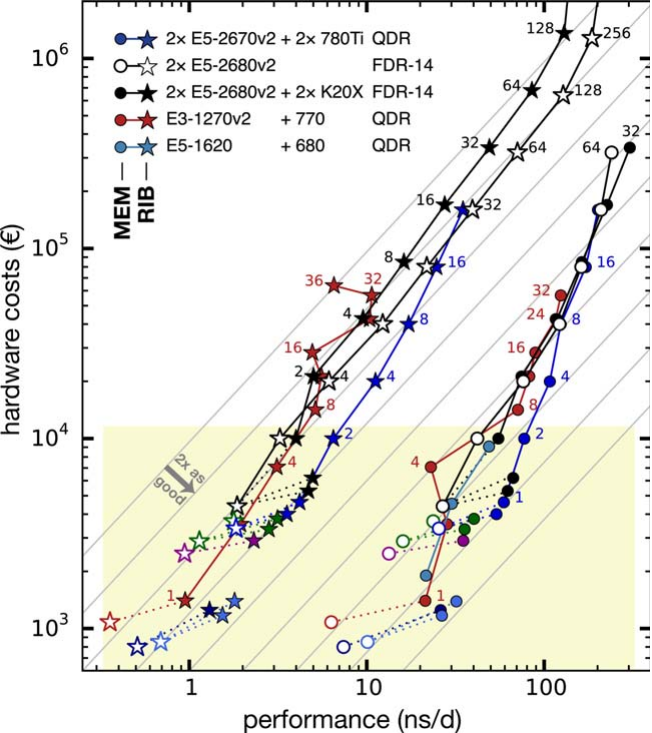
Same representation as in Figure 6 (yellow box depicts section plotted there), now focusing on the parallel performance across multiple nodes (the small number next to the data points indicates the number of nodes used). The gray lines indicate perfect scaling and constant performance‐to‐price ratio, they are a factor of two apart each. A number next to a data point indicates how many compute nodes were used in that benchmark. [Color figure can be viewed in the online issue, which is available at wileyonlinelibrary.com.]

Figures 6 and 7 relate hardware investments and performance, thus, summarizing the results in terms of our criteria performance‐to‐price (C1), single‐node performance (C2), and parallel performance (C3). The gray lines indicate both perfect parallel scaling as well as a constant performance‐to‐price ratio; configurations with better ratios appear more to the lower right. Perhaps not unexpectedly, the highest single‐node performances (C2) are found on the dual‐CPU nodes with two or more GPUs. At the same time, the best performance‐to‐price ratios (C1) are achieved for nodes with consumer‐class GPUs. The set of single nodes with consumer GPUs (filled symbols in the figures) is clearly shifted toward higher performance‐to‐price as compared with nodes without GPU (white fill) or with Tesla GPUs. Adding at least one consumer‐grade GPU to a node increases its performance‐to‐price ratio by a factor of about two, as seen from the dotted lines in the figures that connect GPU nodes with their GPU‐less counterparts. Nodes with HPC instead of consumer GPUs (e.g., Tesla K20X instead of GeForce GTX 980) are, however, more expensive and less productive with GROMACS (black dotted lines).

Consumer PCs with an Intel Core processor and a GeForce GPU in the low‐cost regime at around 1000 € produce the largest amount of MD trajectory per money spent. However, these machines come in a desktop chassis and lack ECC memory. Even less expensive than the tested Core i7‐4770K and i7‐5830K CPUs would be a desktop equivalent of the E3‐1270v2 system with i7‐3770 processor, which would cost about 600 € without GPU, or a Haswell‐based system, for example, with i5‐4460 or i5‐4590, starting at less than 500 €.

Over the lifetime of a compute cluster, the costs for electricity and cooling (C4) become a substantial or even the dominating part of the total budget. Whether or not energy costs are accounted for, therefore, strongly influences what the optimal hardware will be for a fixed budget. Whereas the power draw of nodes with GPUs can be twice as high as without, their GROMACS performance is increased by an even larger factor. With energy costs included, configurations with balanced CPU/GPU resources produce the largest amount of MD trajectory over their lifetime (Tables 8 and 9).

Vendors giving warranty for densely packed nodes with consumer‐class GPUs can still be difficult to find. If rack space is an issue (C5), it is possible to mount 2× Intel E26xx v2/3 processors plus up to four consumer GPUs in just 2 U standard rack units. However, servers requiring less than 3 U that are able to host GeForce cards are rare and also more expensive than their 3–4 U counterparts. For Tesla GPUs, however, there are supported and certified solutions allowing for up to three GPUs and two CPUs in a 1 U chassis.

Small tweaks to reduce hardware costs are acquiring just the minimal amount of RAM proposed by the vendor, which is normally more than enough for GROMACS. Also, chassis with redundant power supply adapters are more expensive but mostly unnecessary. If a node fails for any reason, the GROMACS built‐in checkpointing support ensures that by default at most 15 min of trajectory production are lost and that the simulation can easily be continued.

For parallel simulations (Fig. 7), the performance‐to‐price ratio mainly depends on the parallel efficiency that is achieved. Nodes with consumer GPUs (e.g., E5‐2670v2 + 2×780Ti) connected by QDR IB network have the highest performance‐to‐price ratios on up to about eight nodes (dark blue lines). The highest parallel performance (or minimal time‐to‐solution, C3) for a single MD system is recorded with the lowest latency interconnect. This, however, comes at the cost of trajectories that are 2–8× as expensive as on the single nodes with the best performance‐to‐price ratio.

**Figure 8:**
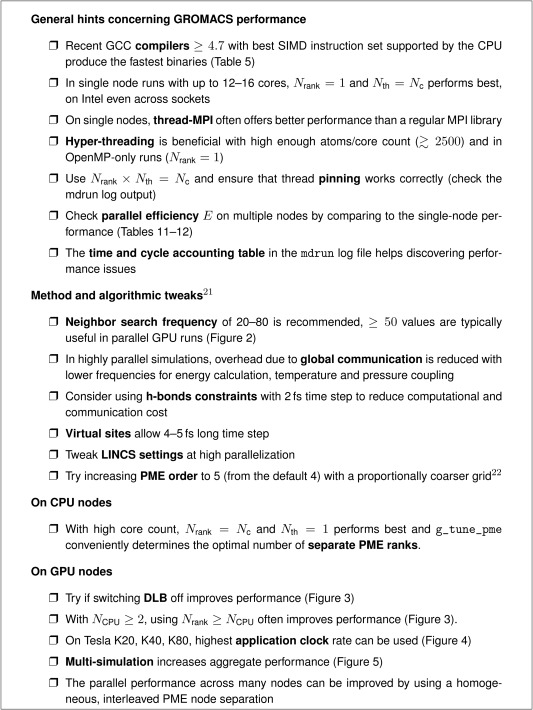
GROMACS performance checklist. Number of MPI ranks, *N*_rank;_ number of OpenMP threads, *N*_th_; number of CPU cores, *N*_c_.

Figure 8 summarizes best practices helping to exploit the hardware's potential with GROMACS. These rules of thumb for standard MD simulations with PME electrostatics and Verlet cutoff scheme hold for moderately parallel scenarios. When approaching the scaling limit of ≈100 atoms per core, a more elaborate parameter scan will be useful to find the performance optimum. Unfavorable parallelization settings can reduce performance by a factor of two even in single node runs. On single nodes with processors supporting HT, for the MD systems tested, exploiting all hardware threads showed the best performance. However, when scaling to higher node counts using one thread per physical core gives better performance. On nodes with Tesla GPUs, choosing the highest supported application clock rate never hurts GROMACS performance but will typically mean increased power consumption. Finally, even the compiler choice can yield a 20% performance difference with GCC ≥ 4.7 producing the fastest binaries.

For the researcher it does not matter from which hardware MD trajectories originate, but when having to purchase the hardware it makes a substantial difference. In all our tests, nodes with good consumer GPUs exhibit the same (or even higher) GROMACS performance as with HPC GPUs—at a fraction of the price. If one has a fixed budget, buying nodes with expensive HPC instead of cheap consumer GPUs means that the scientists will have to work with just half of the data they could have had. Consumer GPUs can be easily checked for memory integrity with available stress‐testing tools and replaced if necessary. As consumer‐oriented hardware is not geared toward nonstop use, repeating these checks from time to time helps catching failing GPU hardware early. Subject to these limitations, nodes with consumer‐class GPUs are nowadays the most economic way to produce MD trajectories not only with GROMACS. The general conclusions concerning hardware competitiveness may also have relevance for several other MD codes like CHARMM,^1^ LAMMPS,^4^ or NAMD,^6^ which like GROMACS also use GPU acceleration in an off‐loading approach.

## Supporting information

Supplementary Information

## Footnotes

1 From version 5.0, the tune_pme command of the gmx tool.

2 Kernel “tail” is the final, typically imbalanced part of a kernel execution across multiple compute units (of a GPU), where some units already ran out of work while others are still active.

3 This is has been verified for version 5.1 which is in beta phase at the time of writing.

4 Although, FFTW supports the AVX instruction set, due to limitations in its kernel auto‐tuning functionality, enabling AVX support deteriorates performance on the tested architectures.

5
http://ipmitool.sourceforge.net/
