# Supplementary material for: Best bang for your buck: GPU nodes for GROMACS biomolecular simulations
Source: J Comput Chem. 2015 Aug 4;36(26):1990–2008. doi: 10.1002/jcc.24030 (PMC5042102; doi:10.1002/jcc.24030)
Supplement: Supplementary file 1 — Supplementary Information [file JCC-36-1990-s001.pdf]

— SUPPORTING INFORMATION —

Best bang for your buck: GPU nodes for GROMACS biomolecular simulations

Carsten Kutzner,[*,†] Szilárd Páll,[‡] Martin Fechner,[†] Ansgar Esztermann,[†] Bert L. de Groot,[†] and Helmut Grubmüller[†]

Theoretical and Computational Biophysics, Max Planck Institute for Biophysical Chemistry, Am Fassberg 11, 37077 Göttingen, Germany, and Theoretical and Computational Biophysics, KTH Royal Institute of Technology, 17121 Stockholm, Sweden

E-mail: ckutzne@gwdg.de

Abstract

This supporting information contains GROMACS exemplary commands to do benchmarks and performance optimization for CPU and GPU nodes.

1 Performance optimization on CPU nodes

1.1 Finding the optimal number of separate PME ranks

To find the best performance-wise settings for a specific `.tpr` file on a node with N_c cores without GPUs, it often suffices to determine the optimal number of separate PME ranks. The largest

[*]To whom correspondence should be addressed

[†]Theoretical and Computational Biophysics, Max Planck Institute for Biophysical Chemistry, Am Fassberg 11, 37077 Göttingen, Germany

[‡]Theoretical and Computational Biophysics, KTH Royal Institute of Technology, 17121 Stockholm, Sweden

benefits are normally seen on nodes with a lot of cores, though also with just a few cores, separate PME ranks may improve performance quite a bit. With the following `bash` commands, the optimal number of PME ranks is determined on a node with 48 cores, using the efficient thread-MPI implementation of `mdrun`.

```
# Add the GROMACS programs to your path:
source /path/to/your/gromacs/bin/GMXRC

# Let g_tune_pme know how the thread-enabled mdrun is called:
export MDRUN=$( which mdrun_threads )

# Invoke the bunch of tuning runs with your .tpr file:
g_tune_pme -ntmpi 48 -s in.tpr -cpt 1440
```

The optional `-cpt 1440` argument sets the interval of checkpoint writing to 24 hours, to suppress unnecessary checkpoints during benchmarking. Please look up other helpful tuning options with `g_tune_pme -h` (for GROMACS 4.6), or `gmx help tune_pme` (for 5.x).

If you need to use a regular MPI library, adapt the following commands to your needs.

```
# Point to the GROMACS bin directory, let g_tune_pme know how the
# MPI-enabled mdrun is called and what command is needed
# to start parallel runs:
source /path/to/your/gromacs/bin/GMXRC
export MPIRUN="/path/to/your/mpi/mpirun -machinefile hosts"
export MDRUN=$( which mdrun_mpi )
g_tune_pme -np 48 -s in.tpr
```

Tuning will produce a table with all successfully tested settings and the resulting performances. For parallel runs across multiple nodes a machine file may be needed depending on the MPI distribution and / or queuing system in use.

Here is an example using IBM's LoadLeveler, which requires that parallel jobs are started using `poe` (not mpirun). Moreover, the number of ranks has to be specified via queue parameters exclusively, and not with `-np` as for other MPI frameworks; this is taken care of by `-npstring none`. In this example intended for 20-core nodes, altogether 640 MPI processes are started on 64 nodes (10 MPI tasks per node), each using two OpenMP threads, thus making use of 20 cores per node.

```
# @ shell=/bin/bash
```

```
# @ job_type = parallel
# @ node_usage= not_shared
# @ node = 64                          # request 64 nodes
# @ tasks_per_node = 10                # start 10 MPI processes per node
# @ resources = ConsumableCpus(2)      # use 2 threads per MPI process
# @ queue
module load gromacs/4.6.7              # e.g.
export MDRUN=/path/to/mdrun_mpi

# This is important so that g_tune_pme knows how to start MPI processes:
export MPIRUN=poe

# Do the tuning runs! Note that -ntomp 2 is not strictly needed here,
# since already specified by the ConsumableCpus(2) line above
g_tune_pme -np 640 -npstring none -ntomp 2 -s in.tpr
```

If one does not want to perform extensive run parameter scans to find the performance optimum, the `mdrun` log file `md.log` still provides useful information on what parameters to change to approach the performance optimum. For example, running the MEM benchmark on the 40 cores of a $2\times$ E5-2680 node using 28 PP plus 12 PME ranks, results in a performance of 20 ns/d. Near the end of the log file, the work balance between the PME mesh part (computed on the PME ranks) and the rest of the force calculation (computed on the PP ranks) is listed, which turns out to be 0.625 in this case.

```
 Average PME mesh/force load: 0.625
 Part of the total run time spent waiting due to PP/PME imbalance: 8.3 %

NOTE: 8.3 % performance was lost because the PME nodes
      had less work to do than the PP nodes.
      You might want to decrease the number of PME nodes
      or decrease the cut-off and the grid spacing.
```

As noted, this is unfavourable as the PME ranks are idle a significant amount of time each step to wait for the PP computations to finish. It should be checked whether performance is increased with a lower number of PME ranks. Using 10 PME ranks indeed yields a performance of about 26 ns/d and a balance very near to 1.0 between PME and PP computation.

```
 Average PME mesh/force load: 1.011
 Part of the total run time spent waiting due to PP/PME imbalance: 0.7 %
```

2 Performance optimization on GPU nodes

For optimal performance on GPU nodes it is necessary to achieve a balanced load distribution between GPU and CPU. How well the load is balanced is obtained from the GPU / CPU force evaluation time listed at the end of the `md.log` output file.

```
 Force evaluation time GPU/CPU: 5.673 ms/8.301 ms = 0.683
For optimal performance this ratio should be close to 1!

NOTE: The GPU has >25% less load than the CPU. This imbalance causes
      performance loss.
```

Here, the automatic tuning of Coulomb cutoff and PME grid spacing was deactivated, resulting in a performance loss due to a too small Coulomb cutoff and too large PME grid, which is clearly disadvantageous.

On the other hand, a very large Coulomb cutoff resulting from automatic GPU / CPU load balancing can be an indicator for a suboptimal hardware configuration. Near the end of the `md.log` file, the values of the Coulomb cutoff and PME grid spacing are listed. The first line provides the input values from the `.tpr` file, the following line the optimized values after GPU / CPU load balancing, here for the example of the 4-GPU benchmark from Table 10 in the main text.

```
 PP/PME load balancing changed the cut-off and PME settings:
           particle-particle                    PME
            rcoulomb  rlist            grid      spacing   1/beta
   initial  1.000 nm  1.012 nm     240 240 240   0.130 nm  0.289 nm
   final    1.607 nm  1.619 nm     144 144 144   0.217 nm  0.465 nm
 cost-ratio           4.10             0.22
```

The fact that the Coulomb cutoff is increased by 60% (or that the GPUs get 4.1 times as much computational load as a result of tuning) indicates a lack of CPU compute power relative to the available GPU power on this node, resulting in decreased performance / Watt.

2.1 Finding the optimal number of threads per rank

On GPU nodes with more than ≈ 8 cores and / or with more than one GPU, you may want to derive the perfect combination of MPI ranks and OpenMP threads (see Figure 3 in the main article). When

the number of ranks on a node does not equal the number of GPUs, a string has to be provided to `mdrun` with the proper mapping of GPUs to ranks. E. g., to use 6 PP ranks with 2 GPUs, the following command assigns GPU 0 to the first three ranks and GPU 1 to the last three.

```
mdrun -ntmpi 6 -gpu_id 000111 -s in.tpr
```

The following `bash` function constructs a `-gpu_id` string based on the number of PP ranks and GPUs and is useful when doing a ranks versus threads parameter scan.

```
#!/bin/bash
# From the number of GPUs per node and the number of PP ranks
# determine an appropriate value for mdrun's "-gpu_id" string.
# GPUs will be assigned to PP ranks in order, from the lower to
# the higher IDs, so that each GPU gets approximately the same
# number of PP ranks. Here is an example of how 5 PP ranks would
# be mapped to 2 GPUs:
#            +-----+-----+-----+-----+-----+
# PP ranks:  |  0  |  1  |  2  |  3  |  4  |
#            +-----+-----+-----+-----+-----+
# GPUs:      |  0  |  0  |  0  |  1  |  1  |
#            +-----+-----+-----+-----+-----+
#
# Will consecutively use GPU IDs from the list passed to this
# function as the third argument.
#
func.getGpuString ( )
{
    if [ $# -ne 3 ]; then
        echo "ERROR: getGpuString needs #GPUs as 1st, #MPI as 2nd, and a"
        echo "string with allowed GPU IDs as 3rd argument (per node)!" >&2
        echo "It got: '$@'" >&2
        exit 333
    fi

    # number of GPUs per node:
    local NGPU=$1
    # number of PP ranks per node:
    local N_PP=$2
    # string with the allowed GPU IDs to use:
    local ALLOWED=$3

    local currGPU=0
    local nextGPU=1
    local iPP
    # loop over all PP ranks on a node:
    for ((iPP=0; iPP < $N_PP; iPP++)); do
        # single char starting at pos $currGPU:
        local currGpuId=${ALLOWED:$currGPU:1}
        # single char starting at pos $nextGPU:
        local nextGpuId=${ALLOWED:$nextGPU:1}

        # append this GPU's ID to the GPU string:
        local GPUSTRING=${GPUSTRING}${currGpuId}

        # check which GPU ID the _next_ MPI rank should use:
        local NUM=$( echo "($iPP + 1) * $NGPU / $N_PP" | bc -l )
```

```
        local COND=$( echo "$NUM >= $nextGPU" | bc )
        if [ "$COND" -eq "1" ] ; then
            ((currGPU++))
            ((nextGPU++))
        fi
    done

    # return the constructed string:
    echo "$GPUSTRING"
}
```

./supplements/benchmarking/constructGpuString.sh

Here is an example for 4 GPUs node, for which the function will create the string ‘`0001122233`’ when requesting 10 ranks per node. On a 40-core node `mdrun` will automatically fill up all available cores by choosing 4 threads per rank.

```
# Example:
USEGPUIDS="0123456789"  # only GPU id's from this list will be used
NGPU_PER_NODE=4
NRANK_PER_NODE=10

# Construct a string that maps ranks to GPUs
GPUSTR=$( func.getGpuString $NGPU_PER_NODE $NRANK_PER_NODE $USEGPUIDS )

# ... and use it!
mdrun_threads -s in.tpr -ntmpi $NRANK_PER_NODE -gpu_id $GPUSTR
```

A parameter scan for the optimal division of cores in ranks and threads $N_{\mathrm{c}} = N_{\mathrm{rank}} \times N_{\mathrm{th}}$ could be done using the following script. Note that GROMACS requires at least one rank per GPU.

```
CORES=40
NGPU_PER_HOST=2
USEGPUIDS="0123456789"
DIR=$( pwd )

# list with all possible numbers of thread-MPI ranks to check.
# Threads will be started automatically by mdrun to fill up all cores
RANKLIST="40 20 10 8 5 4 2"

for NRANK in $RANKLIST ; do
  GPUSTR=$( func.getGpuString $NGPU_PER_HOST $NRANK $USEGPUIDS )
  for DLB in "no" "yes" ; do
    for RUN in ntmpi${NRANK}_dlb${DLB} ; do
      mkdir "$DIR"/run_$RUN
      cd "$DIR"/run$RUN
      mdrun -ntmpi $NRANK -npme 0 -s in.tpr -dlb $DLB -gpu_id $GPUSTR
    done
  done
```

```
done
```

2.2 Separate PME ranks on GPU nodes

If running on multiple GPU nodes in parallel, one might want to assign half of the ranks per node as separate PME ranks. This can be achieved with settings similar to the following, which gives an example using LoadLeveler on 32 nodes with 2 GPUs each. Since the `mdrun` default is an *interleaved* rank assignment, each node will get assigned two PP and two PME ranks. With the `-gpu_id 01` string, these two PP ranks on a node are properly assigned to the two GPUs.

```
# @ tasks_per_node = 4       # 4 ranks per node (2 for PP, 2 for PME)
# @ node = 32                # 32 nodes, i.e. 128 MPI ranks altogether
# @ resources = ConsumableCpus(5)  # use 5 threads per rank for 20 cores

# Use half of the 128 ranks for PME:
poe mdrun_mpi -npme 64 -s in.tpr -gpu_id 01
```

This example is similar to the above one, now using the more common OpenMPI / IntelMPI / MPICH style process startup. Additionally, here the 20 cores are distributed non-evenly across PME and PP ranks to fine-tune the PP-PME compute power.

```
mpirun -np 128 mdrun_mpi -npme 64 -ntomp 4 -ntomp_pme 6 -gpu_id 01 ...
```

2.3 Optimizing throughput with multi simulations

M independent simulations can be started using either the `-multi <M>` or the `-multidir <dirA dirB dirC ...>` option of an MPI-enabled `mdrun`, which automatically takes care of proper process placement and pinning. Note that in case of more than one GPU on a node a proper `-gpu_id` string has to be provided, mapping ranks to GPUs, as given in the following examples for two GPUs and eight replicas. This example uses the `-multi` keyword, in which case the individual `.tpr` input files need to have the replica number attached to the base file name, i. e. here `repl0.tpr`, . . . , `repl3.tpr`.

```
mpirun -np 4 mdrun_mpi -multi 4 -gpu_id 0011 -s repl.tpr
```

Table 1: Enhanced parallel efficiency by running multi-simulations across several nodes, here for the RIB benchmark on 2×E5-2680v2 nodes with 2×K20X GPUs (see lowermost part of Table 12 in the main text). P_1 and E_1 give the performance and parallel efficiency for a single replica densely packed on the available nodes, whereas P_4 and E_4 are recorded for an interleaved placement of 4 replicas on 4 times the number of nodes.

nodes / replica	P_1 (ns/d)	E_1	P_4 (ns/d)	E_4	per replica N_{rank}	N_{th}	
1	3.99	1	3.75	0.94	20	2	ht
2	5.01	0.63	6.56	0.82	80	1	ht
4	9.53	0.6	12.1	0.76	160	1	ht
8	16.2	0.51	20.8	0.65	160	1	
16	27.5	0.43	33.6	0.53	160	4	ht
32	49.1	0.38	55.5	0.43	320	2	
64	85.3	0.33	94.8	0.37	640	2	

Alternatively, with the `-multidir` mechanism, using the run directories `replA`, . . . `replD`, each containing an input file `topol.tpr`, the command is the following.

```
mpirun -np 4 mdrun_mpi -multidir replA replB replC replD -gpu_id 0011
```

`mdrun` will automatically determine the number of cores per node N_c and assign $N_{th} = N_c/M$ threads to each replica. As another option, the number of threads can be controlled with the optional command line parameters `-ntomp` (threads per replica) or `-nt` (total threads), which is useful if performance with and without hyper-threading shall be compared.

The multi-simulation technique can also be used to enhance the performance across several nodes. This requires a fast enough interconnect, as e. g. FDR-14 Infiniband in our test case, see Table 1. Instead of a *dense* placement of replicas, where each replica is spread across as few nodes as possible, each node gets one or more DD domains of each replica. As a result, the replicas are spread across all available nodes in an *interleaved* fashion. Like in a single-node multi-simulation, the individual replicas may run (slightly) out of sync, allowing for better GPU utilization and raised performance. In addition, as the individual replicas perform their communication at different times, less CPU cores compete at the same time for the available network resources as compared to a dense placement. This setup is especially useful for larger MD systems, where running multiple

copies on a single node would be too slow. E. g., when running an ensemble of 4 RIB replicas using 16 nodes one could start 4 completely independent simulations, each using 4 nodes, which would yield a performance of 9.53 ns/d per replica (Table 1). With multi-simulations, the 4 replicas are distributed across the 16 nodes in an interleaved way, which yields 12.1 ns/d per replica and thus a 27 % increased performance.

2.4 Fixing the GPU fan speed

The probability of GPU throttling of consumer GPUs can be diminished by setting the fan speed to a value higher than the default (see Section 2 in the main document). The GPU fan speed can not be set using the `nvidia-smi`; instead, the graphical utility and a running X server is required. First, the `xorg.conf` config file should be modified to include the '`coolbits`' option:

```
Section "ServerLayout"
        Identifier "dual"
        Screen 0 "Screen0"
        Screen 1 "Screen1" RightOf "Screen0"
EndSection

Section "Device"
        Identifier     "nvidia0"
        Driver         "nvidia"
        VendorName     "NVIDIA"
        BoardName      "GeForce GTX TITAN"
        Option         "UseDisplayDevice" "none"
        Option         "Coolbits" "4"
        BusID          "PCI:2:0:0"
EndSection

Section "Device"
[...]
EndSection

Section "Screen"
        Identifier     "Screen0"
        Device         "nvidia0"
EndSection

Section "Screen"
[...]
EndSection
```

Then, start the X server and set the fan speeds:

```
export DISPLAY=:0

/usr/bin/X $DISPLAY -nolisten tcp vt7 -novtswitch &

nvidia-settings -a [gpu:0]/GPUFanControlState=1 \
   -a [fan:0]/GPUCurrentFanSpeed=80

nvidia-settings -a [gpu:1]/GPUFanControlState=1 \
   -a [fan:1]/GPUCurrentFanSpeed=80
```

The fan speed settings can be checked with `nvidia-smi`:

```
+-------------------------------------------------------+
| NVIDIA-SMI 346.47     Driver Version: 346.47          |
|-------------------------------+-----------------------+
| GPU  Name        Persistence-M| Bus-Id        Disp.A  |
| Fan  Temp  Perf  Pwr:Usage/Cap|         Memory-Usage  |
|===============================+=======================+
|   0  GeForce GTX TITAN    On  | 0000:02:00.0      Off |
|  80%   69C    P8   205W / 250W |    549MiB /  6143MiB |
+-------------------------------+-----------------------+
|   1  Quadro M6000         On  | 0000:03:00.0      Off |
|  80%   66C    P0   203W / 250W |    533MiB / 12287MiB |
+-------------------------------+-----------------------+
```
